# Myelin Recovery in Multiple Sclerosis: The Challenge of Remyelination

**DOI:** 10.3390/brainsci3031282

**Published:** 2013-08-28

**Authors:** Maria Podbielska, Naren L. Banik, Ewa Kurowska, Edward L. Hogan

**Affiliations:** 1 Department of Neurology, Institute of Molecular Medicine and Genetics, Georgia Regents University, 1120 15th Street, Augusta, GA 30912-2620, USA; E-Mail: maria.podbielska@iitd.pan.wroc.pl; 2 Department of Neurosciences, Medical University of South Carolina, 171 Ashley Avenue, Charleston, SC 29425-6160, USA; E-Mail: baniknl@musc.edu; 3 Laboratory of Signaling Proteins, Polish Academy of Sciences, Ludwik Hirszfeld Institute of Immunology & Experimental Therapy, Rudolfa Weigla 12, Wrocław 53-114, Poland; E-Mail: kurowska@iitd.pan.wroc.pl; 4 Department of Microbiology, National University of Ireland Galway, University Road, Galway, Ireland

**Keywords:** calpain, central nervous system, demyelination, fingolimod, glycolipids, lipids, multiple sclerosis, myelin, myelination, NKT cells, oligodendrocytes, remyelination, T cells

## Abstract

Multiple sclerosis (MS) is the most common demyelinating and an autoimmune disease of the central nervous system characterized by immune-mediated myelin and axonal damage, and chronic axonal loss attributable to the absence of myelin sheaths. T cell subsets (Th1, Th2, Th17, CD8^+^, NKT, CD4^+^CD25^+^ T regulatory cells) and B cells are involved in this disorder, thus new MS therapies seek damage prevention by resetting multiple components of the immune system. The currently approved therapies are immunoregulatory and reduce the number and rate of lesion formation but are only partially effective. This review summarizes current understanding of the processes at issue: myelination, demyelination and remyelination—with emphasis upon myelin composition/architecture and oligodendrocyte maturation and differentiation. The translational options target oligodendrocyte protection and myelin repair in animal models and assess their relevance in human. Remyelination may be enhanced by signals that promote myelin formation and repair. The crucial question of why remyelination fails is approached is several ways by examining the role in remyelination of available MS medications and avenues being actively pursued to promote remyelination including: (i) cytokine-based immune-intervention (targeting calpain inhibition), (ii) antigen-based immunomodulation (targeting glycolipid-reactive iNKT cells and sphingoid mediated inflammation) and (iii) recombinant monoclonal antibodies-induced remyelination.

## 1. Introduction

Multiple sclerosis (MS) is a chronic disorder characterized by multifocal inflammatory infiltrates (T cells, B cells, macrophages) within the central nervous system (CNS) and with concomitant degradation of myelin, oligodendrocytes and axons, along with reactive astrogliosis and activated microglia [1]. A clinical hallmark is heterogeneous presentation ranging from benign with little or no disability even years after disease onset, a commonly encountered relapsing-remitting course, and the rare fulminant course [2]. Four demyelination pathologies have been recently described suggesting the existence of different mechanisms of inflammation, cell injury and repair [3]. Examining very early lesions however John Prineas and colleagues found few inflammatory cells and therefore challenge the dogma that cellular immune responses are preeminent in MS [4]. Disease progression is thought to be compounded from two underlying processes: myelin destruction (demyelination) with failure to remyelinate [5], and progressive axonal damage with little capacity for recovery [6].

Currently available disease modifying therapies for MS aim to reduce the immune response by targeting immunological pathways: β-interferons, IFNβ-1α (Avonex, Rebif) and IFNβ-1β (Betaseron); the synthetic peptide glatiramer acetate (Copaxone); the antineoplastic agent mitoxantrone (Novantrone), and; a very late antigen-4 (VLA-4) blocker natulizumab (Tysabri) [7], but all are only partially effective. All of these drugs are administered by injection and many MS patients prefer oral treatment [7]. There are three new oral medications, already released and approved by the Food and Drug Administration (FDA): fingolimod marketed by Novartis as Gilenya, dimethyl fumarate (Tecfidera, Biogen Idec, MA, USA), and teriflunomide (Aubagio from Sanofi, Paris, France). These drugs mainly affect lymphocyte trafficking and/or differentiation, though more needs to be done to clarify their mechanisms.

An essential challenge for MS therapy is to target not only the inflammatory aspect of the disease but also its neuroaxonal pathology aiming toward neuroregenerative outcomes. By broad definition, it is an effect that results in salvage, recovery, or regeneration of the nervous system, its cells, structure and function [8]. Here we describe a potential role in remyelination for currently available MS medications, and discuss the many avenues that are being actively studied to promote remyelination. The next frontier aims to delay disease progression and recover lost neurological function via MS therapeutics and eventually develop agents that directly affect myelin repair.

## 2. Myelin Composition and Architecture

**Figure 1:**
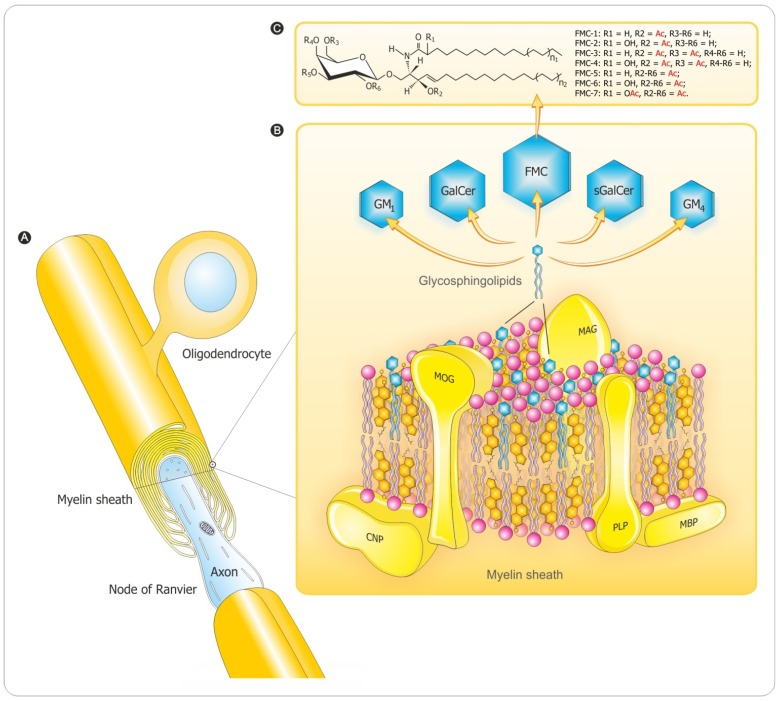
A composite diagram summarizing features of CNS myelin: (**A**) architecture; (**B**) 3D-molecular composition and conformation-based assembly and (**C**) the unique sphingosine 3-*O*-acetylated-GalCer GL series. The diagram depicts arrangement of complex lipids (cholesterol, PLs and GLs) and most abundant proteins (PLP, MBP). The relative molar constancy of lipids: cholesterol (C):PLs:galactosylceramide (GalCer) is C:PLs:GalCer = 2:2:1. Proteins are marked in yellow and the comprising lipids are as follows: cholesterol in orange, PLs in pink and the glycosphingolipids (FMC, fast migrating cerebrosides; GalCer, galactosylceramide; GM_1_, mono-sialoganglioside; GM_4_, sialosyl-galactosylceramide; sGalCer, sulfatide) in blue. Structures of myelin acetyl-cerebrosides (FMCs) are shown. Adapted and modified from Podbielska *et al.* [20] with permission of Future Medicine Ltd. Additional abbreviations: CNP, 2′3′-cyclic-nucleotide 3′-phospodiesterase; MAG, myelin-associated glycoprotein; MBP, myelin basic protein; MOG, myelin oligodendrocyte glycoprotein; PLP, proteolipid protein.

Myelination is an essential CNS developmental process that is characterized by lipid as well as protein assembly into the oliogodendrocyte cell membrane that is elaborated into a spiraling membrane investing axons. The multilayer CNS myelin membrane sheaths surrounding nerve fibers (axons) (Figure 1A) are comprised of lipids and proteins distributed according to charge, lipo- or hydrophilicity, and relative molecular weight [9]. One of the biochemical characteristics distinguishing myelin from other biological membranes is its high lipid-to-protein ratio. The lipids are assembled concomitantly with myelin-specific membrane proteins, the most abundant being the intrinsic (integral) membrane protein—myelin proteolipid protein (PLP) and the extrinsic (peripheral) myelin basic protein (MBP) while myelin-associated glycoprotein (MAG), myelin oligodendrocyte glycoprotein (MOG), and 2′3′-cyclic-nucleotide 3′-phosphodiesterase (CNP) are quantitatively minor constituents (Figure 1B). The three main classes of lipids comprising CNS myelin are cholesterol, glycosphingolipids (derivatives of galactosylceramides (GalCer) and glucosylceramides (GlcCer)), and phospholipids (PLs): these myelin lipid classes have long been known to have constant molar proportions of 2:1:2 [10,11].

Galactocerebrosides (galactosylceramides; GalCer) are the most abundant glycolipid (GL) component of myelin, and constitute a molecular family that differs between members mainly in fatty acid chain length and presence or absence of a fatty acid C2 hydroxyl and with minor changes in the chemical structure of the sphingosine moiety. One-fifth of the total myelin glycosphingolipid constitute sulfatide (sGalCer) with the galactose 3′-OH sulfated: an amphiphatic GL that has a polar sulfated galactosyl head group facing out from the plasma membrane, and anchored in the membrane bilayer by ceramide-containing *N*-acyl-linked long chain fatty acids.

Other derivatives are the GalCer acetylated [12,13,14] or the GlcCer sialylated ones. Seven novel derivatives of GalCer (designated as FMCs for fast migrating cerebrosides on TLC) in vertebrate brain including human have been characterized with complete chemical and structural delineation (Figure 1C). The simplest FMCs (FMC-1 and FMC-2) are 3-*O*-acetyl-sphingosine GalCer incorporating either non-hydroxy or 2-hydroxy fatty acyl attached to the sphingosine base [12]. The next two, FMC-3 and FMC-4, are the 3,6-*O*-acetyl-galactose derivatives [13], and the complex ones FMC-5 and FMC-6 are 3-*O-*acetyl-sphingosine-2,3,4,6-tetra-*O-*acetyl-GalCer with non-hydroxy and 2-hydroxy-fatty *N*-acylceramide types, respectively [14]. The most hydrophobic FMC-7 is the 3-*O*-acetyl-spingosine-2,3,4,6-tetra-*O*-acetyl-GalCer (2-*O*-Ac-HFA) compound. These complex FMCs with penta- and hexa-acetylation of all available GalCer hydroxyls are unique, highly hydrophobic myelin lipids with potential for increased hydrophobic bonding to strengthen myelin membrane lipid interactions, and provoke immune reactions. Minor myelin components (0.1% to 0.3%) include gangliosides—complex *N*-acetylneuraminic acid-containing glycosphingolipids—especially GM_1_ (mono-sialoganglioside) and GM_4_ (sialosylgalactosylceramide) [15].

The other major myelin lipids are cholesterol and phosphatidylethanolamine; much of the latter are plasmalogens with the glycerol 2′-OH fatty acids replaced by an aliphatic long-chain alkenylether. Phosphatidylcholine (lecithin) is a major myelin constituent, and there is less sphingomyelin. Sphingomyelin and cholesterol form membrane domains called “lipid rafts” [16,17] that are sites of such important functions as signal transduction. The CNS lipid composition has been intensively studied in MS in efforts to discern a primary lipid defect [18]. Along with this, the detection of anti-lipid antibodies in MS has been a long-time focus with renewed interest in recent years [19].

## 3. Myelination Process

### 3.1. Oligodendrocyte Differentiation

**Figure 2:**
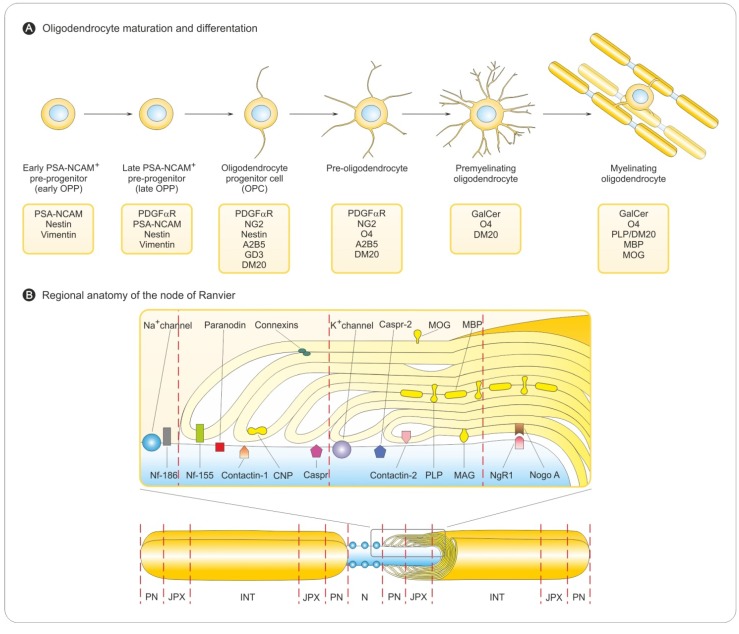
Features of the myelination process. (**A**) Sequential stages of oligodendrocyte maturation. Specific markers for differentiation status of the oligodendrocyte lineage. Differentiation into mature oligodendrocytes is associated with acquisition of myelin-related proteins. (**B**) Myelin sheath organization in the CNS. Myelinating glial cells, oligodendrocytes in the CNS form the myelin sheath by enwrapping axons. Myelinated axon regions are interrupted by non-myelinated regions (nodes of Ranvier). Myelinated axons have four distinct domains: node (N), paranode (PN), juxtaparanode (JXP) and internode (INT). Nodes of Ranvier are regions of concentrated sodium channels that form between two internodes. Adjacent to the nodes of Ranvier are the paranode and the juxtaparanode. All four domains have characteristic proteins (see upper inset). At the nodes of Ranvier, Neurofascin 186 (Nf-186) supports the clustering of Na^+^ channels. The nodal Na^+^ channels are separated from the juxtaparanodal K^+^ channels via the paranode, where Neurofascin 155 (Nf-155) binds tightly to the axonal complex of Contactin and Contactin-associated protein (Caspr). CNP, an abundant cytoplasmic myelin protein, is predominantly found at the paranode. At the juxtaparanode, clustered K^+^ channels are associated to Caspr-2 and Contactin-2.

The central elements of the CNS—myelinating oligodendrocytes and astrocytes as well as neurons—are terminally differentiated cells with a limited capacity to respond to injury. They depend for renewal on the availability of their precursors—the oligodendrocyte progenitor cells (OPCs) and neuronal progenitor cells (NPCs), that undergo proliferation, migration and differentiation into defined progeny [21]. Generation of OPCs in the spinal cord occurs in two phases [22]. The first is ventral, with OPCs observed initially within the ventricular zone of the motor neuron progenitor (pMN) domain of neuroepithelium in early embryonic life [23,24]. Conversely, the second phase occurs in the dorsal spinal cord and hindbrain/telencephalon of the brain in late embryonic development and early post-natal life [25,26,27,28] and depends on inhibition of bone morphogenetic proteins (BMPs) [29]. The proliferating cells migrate into the developing white matter [30,31,32], exit the cell cycle, undergo differentiation into mature oligodendrocytes, and begin to express a subset of myelin-associated proteins [33,34]. Subsequent sequential stages of maturation to myelinating oligodendrocytes are identified by cell type-specific markers (Figure 2A). OPCs are highly proliferative, motile, bipolar cells expressing high levels of the GT_3_ ganglioside, A2B5, platelet-derived growth factor α receptor (PDGFαR), and the neuron-glial antigen 2 (NG2), a transmembrane chondroitin sulfate proteoglycan (CSPG). It is important to recognize differences between developmental myelination and myelin repair in the adult CNS. In the adult brain and spinal cord, OPCs are ubiquitous and represent a large percentage of the total cell population, as much as 9% of cells in white matter and 3% in gray matter [35,36]. Although both processes share many similarities, and the study of developmental myelination contributes to our understanding of myelin regeneration, there are also divergences for the role of transcription and growth factors. Adult OPCs are distinct from perinatal ones [37,38].

### 3.2. Myelin Sheath Organization in the CNS

In the CNS, a single oligodendrocyte can produce up to 40 myelin segments on multiple axons. Consequently, myelinating oligodendrocytes maintain as much as 5–50 × 10^3^ μm^2^ of membrane a day [39]. Maintenance of this myelin membrane occurs throughout adulthood and features a continual turnover of myelin correlated with a high level of expression of myelin genes long after completion of the myelination process [40,41].

Gaps in myelin are caused by the formation of the “nodes of Ranvier”, which are axonal segments where the sodium channels that regulate electrical impulse conduction aggregate (Figure 2B). Myelin also forms the flanking membrane loops, termed the paranodes, which separate the nodal sodium channels from the potassium channels concentrated in the adjacent juxtaparanodal region (Figure 2B). These domains are essential for rapid saltatory conduction. Recent work suggests that axo-glial cell interactions influence the formation of these specialized domains. Both contact-mediated and secreted signaling molecules are involved.

The correlation between the voltage-gated potassium channel (Kv) clusters at the juxtaparanodal junctions with the presence of compact myelin suggests that, in contrast to Nav channel clustering, axonal Kv channel targeting depends on the formation of compact myelin [42].

Reciprocal communication between oligodendrocytes, neurons, and astrocytes plays a key role in the formation of new myelin sheaths [43,44]. Axonal factors needed for different steps in the myelination process can be divided into two groups: those that stimulate, initiate and foster myelination, and others that are inhibitory, and must be removed or blocked for myelination to proceed (*i.e.*, negative regulators of myelination) (see below). There are three putative mechanisms whereby axonal signals regulate myelination [45]: (i) connective with target and ensuing electrical activity: connectivity may influence cell surface expression of adhesion molecules that trigger nearby/associated oligodendrocytes to elaborate myelin sheaths; (ii) activity-induced release of diffusible cues, notably adenosine, that act to inhibit OPCs proliferation and stimulate myelin formation; (iii) direct interactions between OPCs and unmyelinated axons may help regulate the timing of myelination. The balance of stimulatory and inhibitory cues dictates the spatial-temporal orchestration of myelination. Only axons with a diameter larger than 0.2 µm are myelinated in the CNS. Axons become targets for myelination as they start to enlarge [33,46,47]. Furthermore, the number of wraps is precisely related to the axon diameter so that the ratio (called g-ratio) of axon diameter to that of the myelin fiber is constant in normal myelin [48].

### 3.3. Myelination in the Central and Peripheral Nervous System

The myelin sheath plays the same role, and shares several morphological and biochemical properties in both CNS and PNS though there are a multitude of important differences between the myelin sheaths of these two systems, from the embryological and cellular levels to the molecular. They mandate that the two types of myelin be recognized and treated as non-identical [49].

In PNS the transmembrane protein zero (P0) replaces PLP as the major protein (Schwann cells also express low levels of PLP). DM-20, which is identical but shorter than PLP because it lacks 35 amino acids, is also produced from the PLP gene. Low quantities of both PLP gene products are present in the adult PNS, with DM-20 being the most abundant isoform. There are differences in the expression of the PLP gene between oligodendrocytes and Schwann cells because different tissue-specific promoters are used.

In addition to the major P0 glycoprotein, compact PNS myelin contains peripheral myelin protein-22 (PMP22), which accounts for less than 5% of the total protein. Like PMP22, Connexin 32 (Cx32) is an integral membrane protein with four hydrophobic transmembrane domains, mainly found in the paranodal regions and the Schmidt-Lanterman incisures of PNS myelin. Cx32 has been proposed to form gap junctions between different membrane layers of the myelin sheath generated by the same Schwann cell, providing a shortcut for diffusion across the non-compacted regions of the sheath. Another possible role for Cx32 in the PNS is the formation of gap junctions between the myelinated axons and the Schwann cells.

A characteristic of PNS myelin proteins is that at least 60% are glycoproteins. Two high molecular mass glycoproteins are present in relatively small amounts: periaxin and MAG. MAG accounts for only about 0.1% of the total protein in the PNS, and about 1% in the CNS.

Two classes of basic proteins, which are the second most abundant myelin protein group (20% to 30% of the total proteins), are present in the PNS: the MBP and the P2 protein. P2 is a member of a family of fatty acid-binding proteins, with a high affinity for oleic acid, retinoic acid and retinol. P2 may serve as a lipid carrier and could thus be involved in the assembly, remodeling, and maintenance of myelin. Epithelial cadherin (E-cadherin) is also present in small amounts in PNS myelin.

PNS myelin proteins are not distributed randomly throughout the Schwann cell and myelin membranes; some are located in the compact membranes of the myelin sheath (P0, MBP, PMP22), while others are found only in non-compact regions of the sheath (MAG, Connexin 32, E-Cadherin). This spatial distribution cannot be explained simply by differences in protein structure and/or hydrophobicity.

Myelin lipids in CNS *vs.* PNS are qualitatively very similar and only differ quantitatively. Compared to that of other biological membranes, the content of glycosphingolipids with GalCer and sGalCer is high accounting for 14% to 26% and 2% to 7%, respectively, of the total PNS myelin lipid mass in adults. These levels are nevertheless lower than those encountered in the CNS. PNS myelin also contains gangliosides but, here again, the amounts are lower than those encountered in the CNS. In rat sciatic nerve myelin, 90% of the total gangliosides are monosialogangliosides, with sialosyl-lactoneotetraosylceramide (LM_1_) (61%) and GM_3_ (21%) accounting for the large majority. GM_1_ is only a minor constituent of this membrane while GM_4_, which is one of the most abundant gangliosides in brain myelin of some species, is absent from peripheral nerve myelin.

There are more ethanolamine phosphoglycerides (28% to 39%) than those with choline, and plasmalogens (phosphatidylcholine and phosphatidylethanolamine) are abundant in the PNS. Sphingomyelin is more enriched in peripheral nerve myelin, where it represents 10% to 35% of the total lipids, than in brain myelin, where it accounts for only 3% to 8% of the lipids. Phosphatidylserine is not particularly abundant in the myelinated peripheral nerves of mice. There is great enrichment in saturated very long-chain fatty acids (20 to 24 carbon atoms) in myelinated peripheral nerve tissue, and the large majority of these are present in the sphingolipids and, in particular, GalCer. The very long-chain fatty acids are mostly saturated and α-hydroxylated, or non-hydroxylated, and are amidified to the primary amine function of the lipid’s sphingosyl moiety. As in the CNS myelin, the most abundant of the very long-chain fatty acyl groups in adult mouse sciatic nerves is lignoceric acid (C24:0).

It is worth mention that the myelination process in the CNS differs from that for PNS as indicated by Table 1. However there are also similarities, in both CNS and PNS myelin formation is under the influence of progesterone.

## 4. Demyelination Process

### 4.1. Pathology of MS

MS is the most common human demyelinating disease. The prevalent dogma has been that CD4^+^ Th1 cells release cytokines and mediators of inflammation that may cause tissue damage, although CD4^+^ Th2 cells may be involved in modulation of these effects.

**Table 1:** Differences regarding myelination in CNS *vs*. PNS.

CNS	PNS
Myelin sheaths are formed by oligodendrocytes.	Myelin sheaths are formed by Schwann cells (the only glial cell type in peripheral nerves). The myelination of PNS axons by Schwann cells is characterized by the sequential appearance of three different types of nerve fibers: (fetal, promyelin, and myelinated nerve fibers).
Myelination appears when axonal diameter is >0.2 μm.	Myelination occurs only if axonal diameter is >0.7 μm.
A single oligodendrocyte myelinates portions of multiple adjacent axons.	Schwann cells myelinate only one segment of a single axon.
Oligodendrocytes lack plasticity.	Schwann cells have remarkable plasticity.
Glycoproteins are minor constituents of CNS myelin.	Glycoproteins constitute at least 60% of PNS myelin proteins.
Cholesterol required for myelin synthesis is produced by the oligodendrocytes.	Peripheral nerves are hypomyelinated if cholesterol biosynthesis is lacking in Schwann cells [50].
Remyelination requires recruitment and differentiation of progenitor cells into new oligodendrocytes.	Schwann cells can dedifferentiate and assume an immature cell phenotype similar in response to injury.
Limited capacity of regeneration of central axons.	Peripheral axons regrow spontaneously after injury in a permissive environment reflecting the intrinsic regenerative capacity of neurons [51].
Remyelination is regulated by axonal signals that differ for oligodendrocytes and Schwann cells [52,53].	Remyelination is regulated by axonal signals different from those for oligodendrocytes [52,53]. Schwann cells only express a myelinating phenotype when contacting large axons producing threshold levels of neuregulin-1 type III [54].
CNS remyelination can also be achieved by Schwann cells [55] or by immature CNS glia with pluripotent capacity [56].	PNS demyelination reflects Wallerian degeneration and subsequent regeneration [57].

Recent evidence, however, suggests that additional T-cell subsets play a prominent role in MS immunopathology: Th17 cells, CD8^+^ effector T cells and CD4^+^CD25^+^ regulatory T cells. In addition, laboratory and clinical data are accumulating on the prominent role of B cells in MS pathogenesis. This includes a recent paper of potassium inwardly-rectifying channel antibodies with high specificity albeit moderate sensitivity and highlighting the potential effect of monoclonal B cell antibodies on MS prognosis mediating demyelination [58]. Immune cells of both the adaptive and innate systems are involved in the inflammatory network [59,60]. Evidences from experimental autoimmune encephalomyelitis (EAE) studies, as well as the cellular immune response and pathology in MS patients and tissue, suggest the following scenario. The immunopathological events can be divided into: (i) an initial T cell priming, (ii) activation phase in the periphery (*i.e.*, thymus, lymph nodes), and a subsequent (iii) migration of the pro-inflammatory T cells and monocytes across the blood-brain barrier (BBB), (iv) amplification of local inflammation and activation of resident antigen presenting cells (APCs), such as microglia, (v) effector phase of the disease: invasion of CNS parenchyma resulting in damaging of oligodendrocytes, myelin sheath and axons [61]. A possible further stage, the resolution of lesions by regulatory mechanisms and remyelination, is discussed separately (see below).

T cell priming occurs within systemic immune compartments and is initiated by sensitization with myelin antigens including myelin lipids. There are different mechanisms for lipid antigen uptake depending upon the antigen source and its structure such that endogenous lipids are differentially distributed in subcellular compartments and internalized lipids are transported to different endocytotic vesicles depending on the length and unsaturation of their alkyl chains and their mode of internalization [62,63,64]. Lipids with longer alkyl chains are transported to late endosomes [62] corresponding to the localization of CD1b molecules which are specialized for binding long chain lipids [65,66]. Lipids containing alkyl chains that have multiple unsaturation sites or shorter saturated tails are trafficked to early or recycling endosomes [62]; compartments surveyed by CD1c and CD1a that present these types of lipids [67,68].

Antigens presented by APCs within secondary lymphoid organs induce the activation and expansion of myelin-specific T cells, and these activated myelin-reactive T cells circulate through the body searching for their specific antigens to become re-activated [69].

Migration of T cells across the BBB is a complex multi-step process and occurs via interactions between adhesion molecules found on the surface of lymphocytes and endothelial cells [70]. First, circulating T cells slow in the bloodstream due to contact between distinct adhesion molecules on their surface and on endothelial cells. In the second step, homeostatic chemokines, such as CCL19 and CCL21 are produced by cells and mediate T cell activation [71,72], a step followed by third and fourth steps of firm adhesion and final transmigration of the lymphocytes. In several studies the intercellular adhesion molecule-1 (ICAM-1) and the vascular cell adhesion molecule-1 (VCAM-1) expressed on the CNS microvascular endothelial cells and their respective T cell ligands (LFA-1 and VLA-4) are implicated in crucial roles in the transmigration process. In the fifth step, CD4^+^ T cells accumulate within enlarged perivascular spaces where they can encounter their specific antigens (e.g., myelin components) presented by the major histocompatibility complex **(**MHC) class II or CD1 on the surface of APCs such as perivascular dendritic cells [73]. This immune synaptic contact reactivates the T cells. However, for complete activation, differentiation and clonal expansion, a co-stimulating process involving additional molecules is required [74,75]. This antigen-triggered reactivation enables T cells to traverse the glia limitans and migrate into CNS parenchyma.

Upon breaching the BBB, the autoreactive CD4^+^ T cells initiate the local pro-inflammatory cascade. Eventually, a variety of effector mechanisms—including antibody-mediated cytotoxicity, oxygen and nitrogen radicals, pro-inflammatory cytokines and apoptosis-mediating molecules that damage oligodendrocytes, myelin sheaths and occasionally, at this stage, axons—are induced.

Demyelinated plaques and associated astrocytic activation (gliosis) are the results of local inflammation and the major pathological characteristics of the disease [2]. Despite this insight into pathophysiology, the cause of MS remains unclear and definitive treatment of this frequent and chronic disease still eludes us.

### 4.2. Myelin Destruction

**Figure 3:**
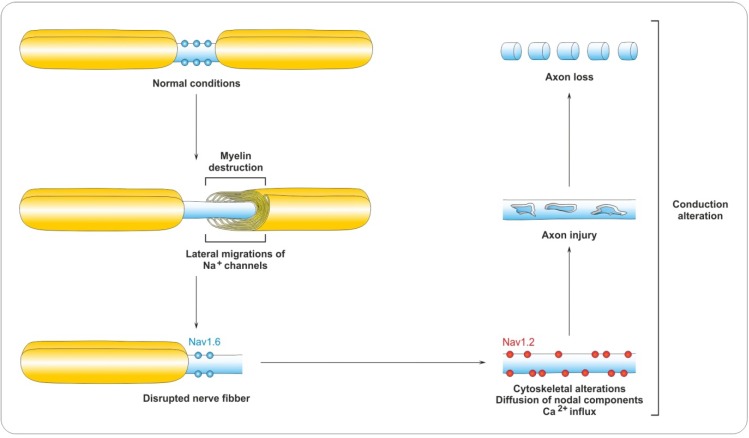
Characteristics of the demyelination process. Demyelination destroys the paranode and sodium channels migrate laterally. Following this is sodium channels (Nav) redistribution and re-expression of the immature isoform of sodium channels, Nav1.2. Persisting currents may cause conduction block and calcium overload, leading to axon injury and eventually loss.

Demyelination in MS is associated with disorganization of paranodal and juxtaparanodal domains. In demyelinated lesions, paranodal (paranodin and contactin-associated protein, Caspr) and juxtaparanodal (Kv channels and Caspr2) proteins become diffusely distributed along denuded axons, and aggregates disappear [76,77]. Early alterations include the overlapping of Neurofascin (Nf)-155-positive paranodal structures with juxtanodal Kv1.2 channels adjacent to actively demyelinating white matter lesions associated with injured axons [78]. Consequently, nodal sodium channels are then directly adjacent to juxtaparanodal potassium channels, leading to impaired saltatory conduction of action potentials (Figure 3). These data suggest that paranodal junctions might be the initial site of demyelination.

The importance of reciprocal communication between axons and oligodendrocytes is apparent in neurological diseases where oligodendrocyte loss and demyelination are associated with a considerable degeneration of axons. Axonal damage and loss of connectivity are clearly critical determinants of disability progression [79,80]. Direct immunologic attack on myelinated axons, from either lymphocytes or soluble inflammatory mediators, accounts for ‘acute’ inflammatory demyelination and axonal loss.

## 5. Remyelination Process

### 5.1. Remyelination as a True Regenerative Process

In MS and EAE, subsequent to the demyelination and degeneration, the opposing effect—remyelination—and neurogenesis are stimulated and progenitor cells migrate into damage sites [21,81]. Remyelination is the default spontaneous process in which demyelinated axons are ensheathed with new myelin sheaths [82,83]. Remyelination is not only found in inactive lesions but can also be observed in lesions with ongoing demyelinating activity [84,85,86]. However, repair processes are especially characteristic of the early disease phase [87]. Prominent remyelination in the early stages of MS has been shown by many groups [84,88], although not invariably reported [89]. Remyelination may even begin within a month or two after active demyelination [84]. In contrast, remyelination in late stage MS appears sparse and restricted to borders of inactive lesions [89,90]. In late stage of MS, remyelination appears limited by oligodendrocyte density, which could be a product of impaired survival, proliferation, and/or migration of oligodendrocytes. Thus, myelin repair, termed remyelination, occurs in acute inflammatory lesions in MS and is associated with functional recovery and clinical remittances. The new myelin sheath may either act as a protective physical barrier to damaging inflammatory molecules [91], or restore trophic support to the axon [92,93,94].

### 5.2. Role of OPCs in Remyelination

It is generally believed that remyelination in the adult CNS is mediated through OPCs identified by the expression of NG2 and PDGFαR [95,96].

Studies using *in vivo* models of demyelination and remyelination, as well as *in vitro* culture systems, have revealed a wealth of knowledge regarding the many sequential and necessary steps involved for OPCs to remyelinate a denuded axon. For remyelination to occur, OPCs must survive, proliferate and migrate to the site of demyelination, driven by chemotrophic factors expressed by either activated microglia or astrocytes within the lesions [97,98]. Once having reached a MS lesion, OPCs must differentiate into myelinating oligodendrocytes as directed by transcription factors, such as Nkx2.2 and Olig2 that are conserved from developmental myelination [99]. Mature oligodendrocytes extend processes toward axons, make contact, enwrap them with concentric layers of myelin membrane, and finally compact these layers into functional myelin [37].

### 5.3. Restoration of the Myelin Architecture and Impulse Conduction

Remyelination is the process of creating new myelin sheaths on axons that have been demyelinated. An immediate consequence of remyelination is proper redistribution of ion channels at the nodes of Ranvier leading to restoration of axonal function by enhancing saltatory conduction [100] and axonal conduction velocity and thereby resulting in functional recovery. Although remyelinated axons appear to regain proper function, there are observable differences in myelin architecture. Most obvious is the thickness of remyelinated sheaths and length of remyelinated internodal segments. Myelin sheath thickness normally increases with axonal diameter, but remyelinated fibers invariably have thinner sheaths around axons of all caliber [101]. Remyelinated internodes also tend to be shorter than developmentally myelinated nodes [102]. New shorter internodes and the thinner new myelin sheaths render remyelinated lesions less intense with myelin stains (so-called “shadow plaques”) [84,103,104,105]. A greater g-ratio remains the most reliable means of identifying remyelinated axons, though with many nodal and paranodal markers, knowledge of internodal length and numbers of nodes may be informative. Remyelinated areas frequently contain lipid-laden macrophages or microglia, which are in contact with thinly remyelinated fibers [84,88] and suggest active cycling between demyelination and remyelination. In contrast to demyelinated lesions, all remyelinated lesions in MS display characteristic aggregates of central Nav channels (nodes), bracketed by paranodin/Caspr (paranodes), Kv channels and Caspr2 (juxtaparanodes) with a mean length, in totally remyelinated lesions that is similar to aggregates found in the periplaque and in control tissue [77]. Furthermore, the length of Caspr, Kv channel, and nodal sodium channel aggregates in shadow plaques is identical to that of normal appearing normal MS white matter. In addition, Nf-186 and MOG appeared to localize appropriately in myelin sheath domains in shadow plaques [78]. Only Nf-155 showed slightly diffuse paranodal mislocalization in remyelinating areas [78], suggesting that Nf-155 localization to paranodes is a late event in remyelination. In partially remyelinated MS lesions, a number of typical triple-Nf-155-positive structures associated with MOG-positive myelin and distinctive unstained nodal gaps have been noted. These represent either transient oligodendrocyte-axonal contacts that occur during the process of internodal remodeling of myelin repair, or aberrant interactions. In shadow plaques, discretely clustered Nav-positive, Nf-186-positive and Nf-155-positive domains indicate restoration of the nodal architecture [78]. Similarly, restoration of normal Nav and Kv channel clusters is observed in remyelination in animal models [106]. More importantly, there is evidence that suggests that demyelinated axons are better protected from subsequent injury when they become remyelinated, perhaps by restoring proper growth factor signaling between the oligodendrocyte and the axon [87,107,108]. Finally, it is likely that the repair process is only possible during a window of time when axons are still intact and myelin competent. Knowledge of the duration of this window, which may vary among different axon types, is crucial for designing therapeutic strategies aimed at promoting appropriately timed myelin repair.

## 6. Regulation of Remyelination

### 6.1. PSA-NCAM

Polysialylated neural cell adhesion molecule (PSA-NCAM) is normally absent from the adult brain but it is re-expressed on the surface of demyelinated axons in MS lesions, and it appears to exert a considerable inhibitory effect on oligodendroglial differentiation [109]. It prevents myelin-forming cells from attaching to axons, which results in a reduced rate of myelination. Conversely, cleavage of PSA from NCAM in co-cultures of oligodendrocytes and neurons substantially increases myelin formation [110] indicating that this posttranslational protein modification is important for restoring neuronal networks with accelerated signal propagation. Down-regulation of the axonal polysialic acid-neural cell adhesion molecule expression coincides with the onset of myelination in human embryonic brain [111]. Similarly, in transgenic mice that exhibit expression of the polysialyltransferase under the control of the PLP promoter prevents down-regulation of PSA in oligodendrocytes, and leads to a reduction in myelin content in the forebrains, both during the period of active myelination and in adult brain [112]. However, in addition to its effect on myelination, PSA-NCAM is needed for OPCs migration [113,114]. The second role, therefore, may complicate the design of strategies aimed at removing barrier PSA to promote myelin repair.

### 6.2. Notch Signaling

The Notch signaling pathway is an important regulator of the balance between OPCs proliferation and differentiation in the developing CNS as well as PNS [115]. The Notch family includes four cell surface receptors (Notch1–Notch4), all of which are type 1 transmembrane proteins. Following binding of the receptor to its ligand, Notch is cleaved intracellularly twice: the first mediated by an ADAM metalloprotease and the second by a γ-secretase complex, releasing an intracellular fragment—the Notch intracellular domain (NICD)—that is translocated into nucleus to activate gene transcription [116]. The ligand engaged on the Notch receptor determines whether the canonical or non-canonical signaling pathway is activated [117]. Both developing and mature oligodendrocytes express Notch 1 receptor [118].

**Figure 4:**
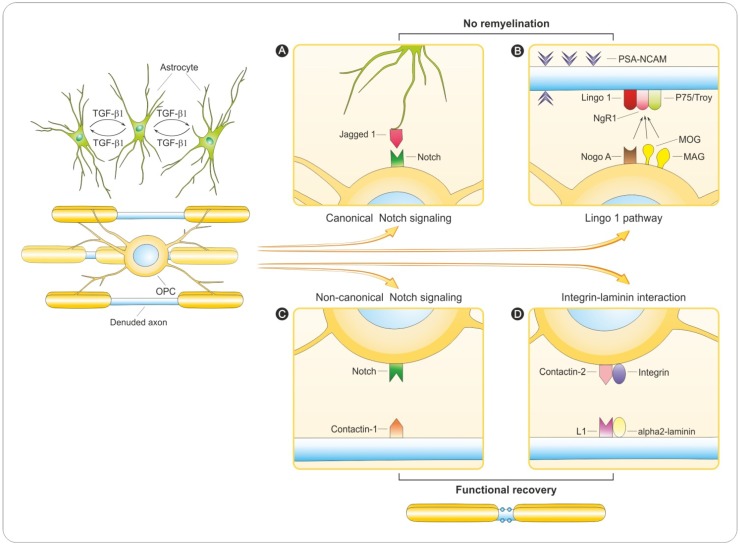
Hypothetical outcomes for demyelinated axon.Denuded axon impermissive for myelination. (**A**,**B**): remyelination failure and axonal death. Oligodendrocyte ensheathing. (**C**,**D**): remyelination leading to long-term neuroregeneration and functional recovery. (**A**) Transforming growth factor-β 1 (TGF-β1), secreted by resident microglial and astroglial cells, stimulates astrocytes in MS lesions to re-express the Notch ligand Jagged1 [121]. Contact-mediated activation of canonical Notch signaling by ligand Jagged 1 inhibits oligodendrocyte progenitor cells (OPC) differentiation and impermissive for proliferation [120]. (**B**) The denuded axon impermissive for myelination through surface expression of inhibitory molecules, such as PSA-NCAM [109], and interactions between axonal LINGO-1 binding to oligodendroglial Nogo-A [133,134,135] prevent further myelination. (**C**) Activation of a non-canonical Notch signaling pathway triggered by axonal ligands including F3/contactin-1 in OPC [123,124]. (**D**) Axonal laminin and L1 bind oligodendroglial integrin (and then dystroglycan receptor) and contactin-2 promoting oligodendrocyte survival and myelination [136]. Additional abbreviations: L1, axonal cell adhesion molecule; MAG, myelin-associated glycoprotein; MOG, myelin oligodendrocyte glycoprotein; NgR1; Nogo-receptor 1; OPC, oligodendrocyte progenitor cell; PSA-NCAM, polysialic acid-neural cell adhesion molecule.

Many factors involved in MS are expressed during development and are re-expressed or show increased activity in the disease state. This is particularly true for Jagged 1, one of five ligands of the Notch receptor. Jagged 1 can be expressed by axons, neurons and astrocytes throughout the brain [119]. In chronic active MS lesion, astrocytes were shown to express Jagged 1, where they are thought to interfere with efficient differentiation of OPCs and remyelination. Surprisingly, in adult cuprizone-treated transgenic mice lacking Notch in PLP-expressing oligodendrocytes (PLP-creERNotch^lox/lox^ mice), conditional ablation of Notch 1 in oligodendrocytes did not produce a marked effect on remyelination [119]. Nevertheless, in a different study [120] Notch 1 inactivation was achieved using conditional gene expression system Olig1-Cre and Notch 1 floxed allele (Olig1Cre:-Notch1^12f/12f^). In these mice oligodendroglial differentiation was accelerated during development and myelin appeared to be normal. Remyelination after lysolecithin-induced demyelination has also been examined and more remyelinated sheaths were observed. In addition, an *in vitro* myelination experiment utilizing Notch 1 siRNA confirmed that Notch 1 signaling was permissive for OPC expansion but inhibited differentiation and myelin formation [120]. These studies revealed that astrocytes exposed to TGF-β1 restricted OPCs maturation, suggesting that canonical Notch 1 signaling is involved in adult CNS remyelination (Figure 4A). It should be mentioned that TGF-β1 induces expression of Jagged 1 in reactive astrocytes [121]. Moreover, this upregulated cytokine in MS was expressed by microglia and astroglia in demyelinating MS lesions [122] whereas Jagged 1 was expressed by reactive astrocytes in demyelinated but not remyelinated lesions. Taken together, both *in vitro* and *in vivo* approaches link Notch signaling with oligodendrocyte differentiation and myelination [118,120], having a particularly powerful inhibitory effect on oligodendrocyte differentiation. These results suggest that Notch might be critical for achieving remyelination and that Notch could be a therapeutic target in myelin disorders.

Another facet of Notch signaling is via non-classical ligands members of the contactin family. Both contactin and NB-3 promote oligodendrogenesis and OPCs differentiation [123]. Contactin 1 (known also as neural cell surface protein F3) promotes the differentiation of oligodendroglial cells *in vitro* and up-regulates MAG expression in oligodendrocytes [123,124]. Because contactin is normally expressed at the axonal paranode, unmyelinated axons receptive to myelination may upregulate contactin to signal oligodendrocytes to mature and begin myelination (Figure 4B). Furthermore, contactin was abundantly expressed in demyelinated axons in human chronic MS lesions [88]. This is of particular interest because contacin-2 has also been identified as an autoantigen targeted by T-cells *in vivo* [125], and contactin binding is able to induce γ-secretase-mediated nuclear translocation of NICD, which is required for myelination signal transduction. In MS, this particular promyelination signal can be attenuated by the induction of TIP30, an inhibitor of Importin β [88]. Consistent with this, expression of TIP30 blocked contactin dependent expression of MAG and cellular differentiation *in vitro*. In addition, TIP30 inhibits differentiation by preventing NICD translocation.

In summary, Notch 1 activation might depend on both the expression of astrocytic and axonal ligands, either of which could promote or inhibit oligodendrocyte differentiation. Overall, the role of Notch 1 in remyelination in MS becomes more complicated over time. In EAE, Notch 1 appears to block remyelination, although it is unproven whether Jagged 1 is the responsible Notch 1 ligand. Studies of MS tissue show that Notch 1 signaling is blocked by TIP30 up-regulation. Given the opposing roles observed for canonical and non-canonical bidirectional function of Notch signaling, it remains to be clarified how this important pathway can be modulated or supported in order to fully enhance remyelination.

### 6.3. LINGO-1 Pathway

Injured adult CNS axons, in contrast to PNS, are unable to regenerate due to inhibitory factors resulting in loss of CNS functions leading to progressive disabilities [126]. Five myelin-associated inhibitory factors (MAIFs) have been identified: MAG [127], MOG [128], Nogo-A [129], Sema4D/CD100 [130] and ephirin B3 [131]. MAG, MOG and Nogo-A bind a three component receptor complex of NgR1, p75 and LINGO-1 to activate the RhoA pathway that inhibits axon regeneration. NgR1 is a member of the leucine rich repeat (LRR) superfamiliy of molecules [132]. LINGO-1 (leucine rich repeat and Ig domain containing 1) also belong to LRR family [133] while the NGF (nerve growth factor) receptor p75 is a member of tumor necrosis factor (TNFR) superfamily.

Functional diversity of p75 is attributed to its ability to interact with different protein partners to form multimeric receptor signaling complexes [137,138]. As a complex with Trk receptors, p75 regulates neuronal survival and axon growth upon binding to neutrophins [139]. As a complex with sorticin, p75 induces neuronal cells to undergo apoptosis upon binding to pro-NGF [137]. As a complex with NgR1 and LINGO-1, p75 inhibits axon regeneration upon NgR1 binding to myelin components [138]. RhoA activation requires proteolysis of p75 by secretases after forming a complex with NgR1/LINGO-1 [140]. P75 in this complex can be functionally replaced by the another TNF family receptor, broadly expressed in postnatal and adult neurons, Troy [135].

LINGO-1 is expressed in normal CNS, specifically by neurons and oligodendrocytes [133]. LINGO-1 expression was also investigated in MS brain [133,134,141] and it was found that total protein expression is reduced, whereas TROY is up-regulated. In MS lesions LINGO-1 has been identified only in reactive astrocytes, macrophages/microglia and neurons but not in oligodendrocytes [141]. LINGO-1 blocks oligodendrocyte differentiation, MAG and MBP expression and myelin formation *in vitro* in a dose-dependent manner [134,139]. Inhibition of LINGO-1 function enhances remyelination in EAE animal models [142].

These results suggest that the axonal complex Nogo receptor NgR1, Troy/p75 and LINGO-1 might modulate neuro-glial interactions in demyelinated lesions by interacting with Nogo-A expressed on oligodendrocytes and this interaction may prevent oligodendroglial maturation and, hence, remyelination [45] (Figure 4C). Evidence from both *in vitro* and *in vivo* studies indicates that anatagonizing LINGO-1 favors both neuroprotection and remyelination, making LINGO-1 an exciting candidate for therapy [142]. The studies have provided a basis for treatment of MS through blocking LINGO-1 function [143]. In closing, a phase 1 clinical trial evaluating the safety and potential therapeutic utility of anti-LINGO-1 mAb in MS has already been completed (Clinical_Trials.gov identifier: NCT01244139).

### 6.4. Integrin-Laminin Interaction

A key step in the process of development and maturation of oligodendrocytes is regulation by axon-glial interaction. This is particularly true for interactions of oligodendroglial cell-derived integrin with axon-derived laminin that contribute to myelination. Integrin links extracellular environments with intracellular signaling molecules. Both α6 and β1 integrin knockout mice show increased oligodendrocytes death during development, and integrin binding to laminins, present in the extracellular matrix surrounding axons, increases survival at low growth factor concentrations. Oligodendrocytes cultured on laminin-2 enhance myelin sheaths and mice and humans that lack the lamininα2 chain are dysmyelinated. On the other hand oligodendrocytes deficient for β1 integrin are unable to ensheath axons, highlighting a role for β1 integrins in fostering early phases of myelination.

In the CNS, laminin and integrin β1 activate the pro-myelinating signals Akt-1 [144], Fyn [136,145,146] and p38 MAP kinase [111]. Recently Laursen *et al*. proposed the existence of an integrative signaling unit composed of α6β1 integrin and its novel partner contactin [a glycosylphosphatidylinositol (GPI)-anchored cell adhesion molecule of the immunoglobulin family] interacting with axonal cell adhesion molecule L1 and laminin [136] (Figure 4D). In this case signals from soluble growth factors, extracellular matrix and cell adhesion molecules on the axonal surface are integrated to enhance oligodendrocyte survival. L1 is expressed on axons in developing CNS with a peak expression at the onset of myelination. Increased cell number and higher percentage of MBP-positive cells myelinate axons in L1-Fc treated cultures [136]. The integration of axonal signals from L1 and laminin present in the extracellular matrix and which binds to contactin present on the surface of oligodendrocytes achieving a novel complex of contactin/integrin through Fyn activation [136]. The presence of L1-Fc, the extracellular portion of contactin ligand expressed on axons not only enhanced survival but additionally promoted myelination in co-cultures of neurons and oligodendrocytes [136]. In addition to this involvement in the myelination process, axonal contactin, initially diffusely distributed along the axons, later aggregates at paranodal junctions, where it interacts with Caspr and Nf-155 [147].

Laminins are also recognized by “non-integrin” receptors including dystroglycan. For the CNS, blocking antibodies to dystroglycan receptors did not affect oligodendrocyte survival but did affect oligodendroglial production of complex membrane sheets and myelin segments when added on dorsal root ganglia neurons *in vitro* [148] suggesting that dystroglycan receptors may have a specific role in the regulation of terminal stages of myelination. Taken together, these results imply that axonal laminins first signal initiation of myelination through integrin/contactin receptors and then to myelination completion via dystroglycan receptors.

### 6.5. Why Does Remyelination Fail?

Although in adult human brain remyelination has been demonstrated in MS lesions [149,150], the capacity for regeneration in MS appears limited [151]. The mechanisms underlying this failure of repair are incompletely characterized [152,153]. There are a multitude of hypotheses as to why remyelination fails in MS, which may reflect either changes in environmental inputs or intrinsic pathways regulating OPCs functions [154,155]. Several factors are likely to impair the completion of remyelination. Among them are factors related to a defect in OPCs activation and recruitment, or to local inhibitors of remyelination [45,156]. Theoretically remyelination can be blocked at any point in the remyelination process: oligodendrocyte survival, proliferation, migration, maturation, and/or myelin sheath formation. In MS lesions late in the course of disease, oligodendrocyte recruitment is deficient and appears to be the primary reason for poor remyelination in late stage MS [153]. In late stage MS, remyelination appears limited by oligodendrocyte density, which could be a product of impaired survival, proliferation, and/or migration of oligodendrocytes. In lesions containing more oligodendrocytes, impaired oligodendrocyte maturation is a major problem for efficient remyelination of lesions. Beyond the oligodendrocyte recruitment and maturation, myelination also requires contact between axons and oligodendrocytes and creation of multiple wraps of oligodendrocyte processes around the axon, culminating in the myelin sheath. Another factor is that repeated demyelinating insults, as observed in the relapse-remitting form of MS, can exhaust the OPCs source so that remyelination failure may be regionally defined due to exhaustion of distinct progenitor pools. Also the glial scars formed within the lesion likely serve as a barrier between inflamed damaged tissue and normal brain and the glia are thought to secrete factors that may inhibit OPCs migration into the scar and subsequent differentiation. The presence of myelin-associated debris in the glial scar, including MAG, MOG and Nogo-A can prevent axonal regeneration and inhibit OPCs differentiation as well [157]. An additional hypothesis for the failure in remyelination in MS proposes that re-expression of developmental regulators of myelination, such as LINGO-1 and PSA-NCAM, that may be re-expressed on stressed axons within the lesion and repel OPCs [158] (see above). The slow rate of OPCs repopulation of demyelinated lesions observed in animal models has led to concerns that remyelination may be unsuccessful in MS due to a temporal discordance between OPCs migration into the lesion and the inflammation required for OPCs activation that is generated from myelin breakdown [159]. Thus, there are a number of possible issues that limit remyelination within MS lesions. These include most prominently problems with oligodendrocyte maturation and the formation of myelin sheaths. Other significant factors include impaired oligodendrocyte migration and/or proliferation in lesions.

Interestingly, recent studies have suggested that remyelinating cells may also arise from the proliferation and differentiation into mature oligodendrocytes of NPCs from germinal areas of the telencephalon such as the subventricular zone [160]. Thus NPCs may represent an additional source of remyelinating cells in MS. Like all progenitors, OPCs and NPCs are subject to precisely coordinated intrinsic and extrinsic signals that provide highly regulated control of the balance between and timing of proliferation and differentiation. Manipulation of these signals may facilitate expansion of the available pools of progenitors, and/or their subsequent differentiation into myelin-forming cells.

## 7. Cytokine-Based Immuno-Intervention

### 7.1. Inflammation and Repair in MS under Cytokines Control

Cytokines are essential mediators of the immune response, and an imbalance in the cytokine network plays an important role in the initiation and perpetuation of autoimmune diseases. There are multiple pathways in which cytokines are involved in MS pathology. In the immune compartment, cytokines are involved in modulating APCs for ideal antigen presentation to CD4^+^ T cells via the up-regulation of MHC class II molecules [161]. Additionally, cytokines stimulate APCs to up-regulate costimulatory molecules required for full T cell activation [161,162]. During T cell priming, APCs also release cytokines that induce differentiation of naive CD4^+^ T helper cells into effector or regulatory T cell subsets: Th1, Th2, Th17, or regulatory T cells (Treg) [162]. Cytokines are also involved in aiding T cell trafficking into the CNS [163,164]. In the CNS compartment, cytokines affect the permeability of the BBB [165,166], OPCs differentiation [167], and activation of microglia and astrocytes to participate in disease progression and remyelination [168].

The activation of myelin-specific T cells accompanied by dysregulation of Th1, Th2, and Th17 cytokines is thought to be a major event for MS initiation, disease development and progression [169]. Production of pro-inflammatory Th1 cytokines (e.g., TNF-α, IL-2, and IFN-γ) by CD4^+^ T cells is increased in MS patients during an exacerbation [170]. In contrast, anti-inflammatory Th2 cytokines (e.g., IL-4, IL-10, and IL-13) predominate during disease remission [171]. The contribution of IL-17 producing Th17 to autoimmune pathogenesis is a current focus of MS research. A recent study concluded that IL-6 selectively promotes the proliferation of Th17 cells by activating the T cell gp130–STAT3 pathway [172]. Since IL-6 exerts only a minimal effect on Treg development, a drug that effectively blockades the IL-6–gp130–STAT3 pathway in CD4^+^ T cells could inhibit harmful Th17-mediated effects. The development of Th17 cells may depend on the presence of IL-23 during antigen stimulation [173], where IL-23 has a key role in Th17-inflammation *in vivo* [174]. Moreover, recent data suggest that human Th17 differentiation is under mediated control of TGF-β-enhanced responsiveness to IL-23 [175]. In MS, increased numbers of peripheral blood mononuclear cells (PBMCs) have been shown to express high levels of IL-17 mRNA, particularly during exacerbations [176]. In summary, cytokine-based manipulation offers a unique possibility to interfere with autoimmune diseases, including MS. However, the potency of cytokines coupled to the complexity of the cytokine network can lead to severe side effects, which can still occur despite careful preclinical evaluation.

### 7.2. Anti-Inflammatory Effect of Calpain Inhibition in MS

The increased influx of calcium ultimately results in activation of multiple enzymatic processes [177,178]. While the events leading to pathophysiology in MS are not clearly understood, proteases, specifically the Ca^2+^-dependent neutral protease—calpain, may play an important role. Activation and expression of calpain is observed in various neurological disorders including MS and its animal model, EAE. The calpains are a family of at least 15 nonlysosomal, intracellular cysteine proteases that are activated by Ca^2+^ at neutral conditions in cells [179]. Both ubiquitous and tissue-specific calpains have been identified. Ubiquitously expressed ones exist in CNS in two forms: as μ-calpain and m-calpain requiring μM and mM Ca^2+^ concentrations for activation, respectively [180], and are regulated by calpastatin (its endogenous inhibitor), lipids and activator proteins [181,182]. Calpains play roles in many molecular processes including cell proliferation and differentiation, T cell activation [183], immune cell migration [184], signal transduction [185], necrosis [186] and apoptosis [187].

Increased activity and expression of calpain have been detected in CNS tissue, immune cells of MS patients and in its corresponding animal model, EAE [187,188,189]. During the active stage of MS pro-inflammatory Th1/Th17 cells predominate over immuno-regulatory Th2/Treg cells [190]. We have shown that increased calpain activity is correlated with Th1/Th2 cytokine dysregulation in MS patient PBMCs during both relapse and remission and that calpain inhibition in these cells attenuates secretion of IL-2 and IFN-γ, thus promoting an anti-inflammatory cytokine bias [191]. We have also examined the effect of calpain inhibition upon expression of Th1/Th17-associated cytokines and their mRNA and protein levels in MS patient PBMCs. We found that calpain inhibition downregulated several inflammatory cytokines (IL-6, IL-12, IL-17, IL-23, TNF-α and G-CSF) while it up-regulated indoleamine 2,3-dioxygenase (IDO) expression; an enzyme that can lead to starvation and stress of Th1 cells, impaired function of bystander Th1 cells and immune cell apoptosis. We think that calpeptin treatment could produce inhibitory factors such as IDO that suppress T cell activation alleviating disease severity. In response to calpeptin or recombinant IDO, the proliferation of T cells was significantly attenuated. The role of IDO in promoting Th2 type cellular immune responses is supported by the data from the EAE model induced by adoptive transfer of MBP-specific T cells, in which both IDO mRNA expression and the kynurenine to tryptophan ratio, which reflect IDO activity, are increased during the remission phase. Reductions in Th1 immune function through increased IDO production could further attenuate inflammatory cytokines, chemokines and alleviate neuthrophil migration, hallmark of MS development and progression. Thus, a high level of IDO expression may precede a favorable shift in Th1/Th2-mediated immune responses in the chronic inflammatory disease MS. These results point to an ability of calpeptin to reduce the pro-inflammatory cytokine profile of MS PBMCs, a feature that could reduce disease severity and prolong patient remission time [192].

In the acute Lewis rat model of EAE, calpain expression was correlated with T cell, macrophage, and neutrophil migration into the CNS [193]. Examination of the acute EAE spinal cord revealed that Ca^2+^ influx and calpain expression correlated with axonal damage, mitochondrial damage, and loss of structural integrity of microtubules and filaments [187].

It was recently reported that the administration of the calpain-inhibitor, CYLA (cysteic-leucyl-argininal), in chronic progressive EAE (MOG-induced disease model of MS) resulted in barely detectable levels of axonal breakdown, as measured by amyloid precursor protein levels [194]. However the preclinical evidence that indicates a beneficial effect of calcium channel blockers is enticing although there are as yet no extant clinical trials of calcium channel blockers in patients with MS.

In summary, our group has identified a role for calpain in the pathogenesis of MS and EAE, including demyelination [193], axonal damage, loss of neurons and oligodendrocytes [187] and modulation of proteins involved in apoptotic pathways [195]. In addition, calpain inhibition has been shown to attenuate the immunogenicity of MBP-specific T cells and disease severity in EAE, including the relapsing/remitting (R/R) model of the disease [187,196,197]. These studies indicate that inhibition of calpain may ameliorate immune pathology in MS. More importantly they implicate the use of calpain as a target for disease modification. Investigating the important role of calpain and its inhibiton on T cell cytokines and chemokines as well may provide valuable insight into the mechanisms by which CD4^+^ T cells elicit neurodegeneration in demyelinating diseases and may lead to novel therapies for MS.

## 8. Antigen-Based Immune-Intervention

### 8.1. Implications of Lymphocyte GL Reactivity and Anergy for Myelin Repair

The three mechanisms [198] contributing to control of T cell reactivity are: (i) deletion; the physical elimination of antigen-specific T cells, (ii) anergy; the lack of T cells antigen responsiveness, and (iii) suppression; the inhibition of T cell function by a regulatory (suppressor) T cell. Lymphocyte roles can be revealed by changes in T cell reactivity and failure of these mechanisms may foster autoimmune diseases. Interference by inflammation-related alterations may affect remyelination. We postulate that for innate reactivity NKT cells regulation are relevant as for instance by treatment with the glycosphingolipid α-GalCer that elicits a NKT-cell-mediated suppressive effect on the effector function of encephalitogenic T cells [199] while for adaptive immune response CD4^+^ and CD8^+^ regulatory T cells can suppress EAE and their induction or enhancement influence remyelination regulation. Circulating lymphocytes in MS [200,201] are hyporesponsive to specific antigens. Importantly, these antigens are certain GLs that have been identified as exogenous but are almost certainly endogenous as well. The robust and GL-specific altered innate immune response in MS suggests that a completed robust iNKT activation with potent cellular and cytokine activities had occurred in the generation of the inflammatory demyelination. It may be that diverse GLs including the endogenous myelin acetylated-galactosylceramides (FMCs) can drive activation critical to controlling CNS inflammation and fostering myelin repair. It should be emphasized that iNKT cells and their invariant or iTCR (Vα24Jα18Vβ11) receptors constitute an innate defense separate from the T and B cell mediated adaptive immunity. The invariant NKT cells are part of a discrete immune arm separate from peptide-driven acquired immune responses.

The immunogenic lipids—complex GLs and sphingolipids—are unlike peptides that reside internally in that complex lipophilic molecules are located on the cell surface and therefore first encounter host immune defenses. Thus, they are situated to enable breaking tolerance with autoimmune reactivity, inflammation and disrupted myelin regeneration. The mechanism of intolerance is problematic but molecular over-lapping implicit in molecular mimicry [202] or polyspecificity [203] is a plausible means for a protective defensive immune response to pathogen- or danger-associated molecular patterns (*i.e.*, PAMP or DAMP conformations) to generate self-destructive attack or inhibition of remyelination. It’s a practical reality that the slow pace and neglect of research pursuing lipids as driving antigens stems from their hydrophobic nature with solubility barriers complicating assays because the lipid limited aqueous solubility affects antigen presentation as well as binding reproducibility and sensitivity.

Targeting myelin lipids as molecules of interest is in line with their important roles as immunogens and bioactive mediators [19,20,204,205] along with their recognized roles in a layered membrane investing axons and facilitating saltatory conduction. Myelin is greatly enriched in complex lipids and sterols as discussed above and depicted in Figure 1B. All of them are potent immunogens and can affect inflammation.

**Figure 5:**
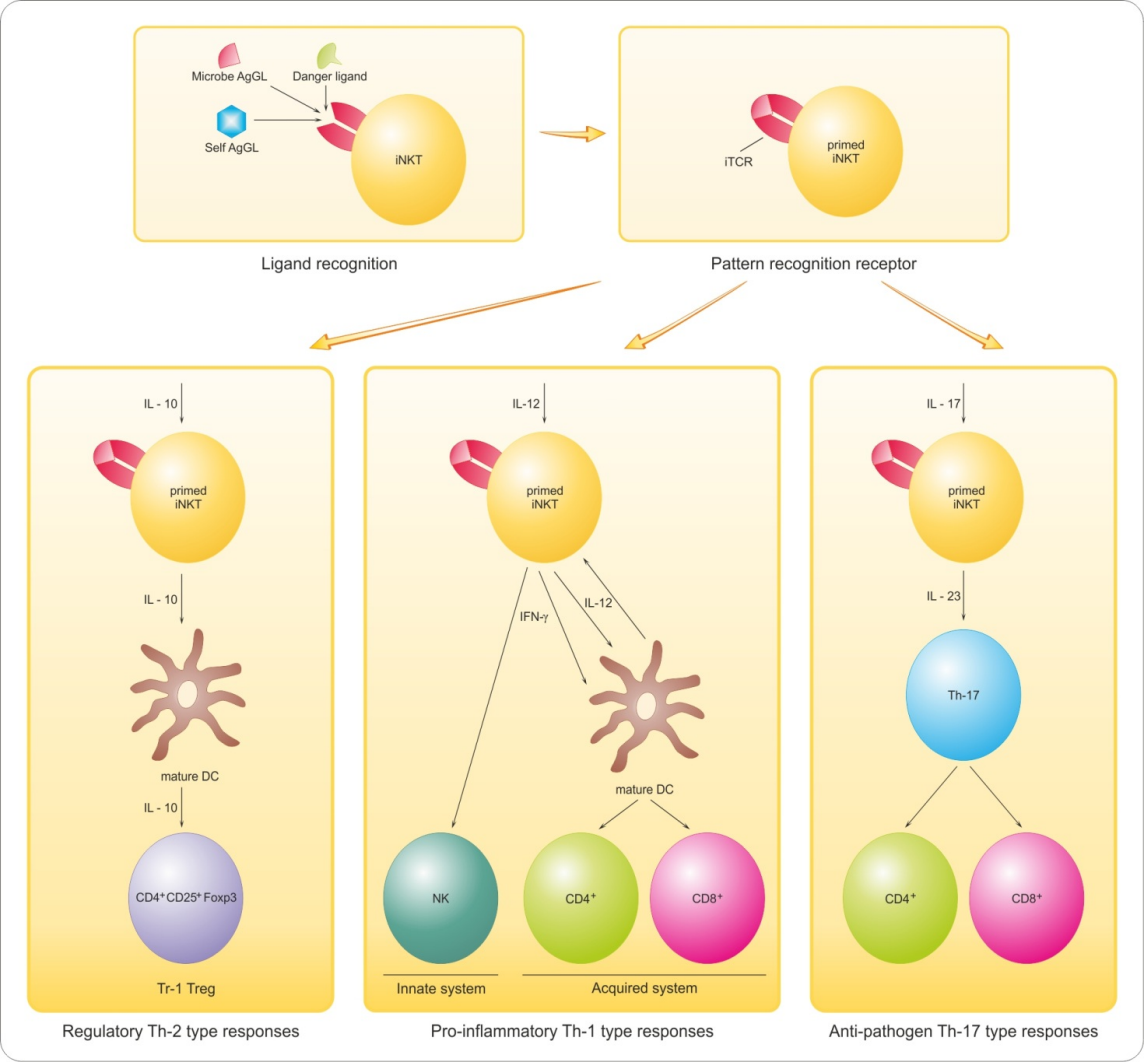
INKT cell-mediated immune responses (IR). Different antigenic GLs: microbial GLs; danger ligands; self antigens (Ags) compete for binding to iTCR; a pattern recognition receptor (PRR). Recognition is for different lipids—e.g., GLs, PLs and IR can be to microbial GLs (usually with high affinity) and overlap IR to self Ags (weaker affinity). The iNKT is (1) primed by the GL/iTCR/CD1d receptor complex and (2) responds according to environment, mainly cytokines-dependent with diverse outcomes: (i) pro-inflammatory (Th-1 type responses), (ii) regulatory (Th-2 type) or (iii) anti-pathogen (Th-17 type) as shown for IL-12 for iNKT pro-inflammatory functions; for an IL-10-driven response generating regulatory Tr1 Treg (e.g., CD4^+^CD25^+^Foxp3) and also anti-pathogen activity triggered by IL-17 and IL-23. From Hogan *et al.* [210] with permission of OMICS Publishing Group.

Several types of regulatory cells including CD8^+^ T cells, B cells and NKT cells participate in controlling pathological autoimmunity (see review [19]). NKT cells compel interest because invariant NKT cells are not only GL-reactive but also as described above constitute a distinct potent arm of the immune system that is separate from conventional peptide-binding T cells [206]. The invariant NKT (also designated as iNKT or type I NKT) cells employ a single species of TCR encoded by Vα24Jα18 α-chain gene segments in humans [207]. This iTCR binds mainly GL antigens and requires for lipid presentation CD1d; a non-classical monomorphic MHC-related antigen-presenting-cell (APC) surface protein [207]. The breadth of molecules reacting with the iNKT receptor (the iTCR) defines the iTCR as an innate pathogen pattern recognition receptor (PRR). The crystal structure of the complex formed by the potent ligand α-GalCer, human CD1d and the iTCR (Vα24Vβ11) has been examined and binding sites for both the GL ligand and the CD1d presentation module identified on the iTCR α-chain [208]. Both iTCR and CD1d are conserved over evolutionary distance emphasizing the importance of NKTs in humans. The invariant TCR binds many GLs [209] that serve to activate and prime the iNKT cell for subsequent activation mainly by cytokines including IL-12, IL-10 and IL-17 for diverse roles including inflammation and regulation. The two-step priming and subsequent activation by different molecules must be emphasized in this process. The second stage or immune activation responses include: (i) Th1-biased inflammation, (ii) regulatory T cell maturation, and (iii) pathogen defense [206]. These events are depicted in our scheme (Figure 5) outlining GL ligand/iTCR binding, subsequent T cell priming and activation to different functions that is determined by the local tissue environment and especially cytokines.

INKT cells are cytokine-rich, potent and versatile, and the iNKT cell interface with innate GL conformational signal is a transformational bridge able to elicit specific cytokine messaging by IL-1, TNF-α, IL-12, IL-17 and others en route to specific responses by the acquired immune system. INKT cells produce large amounts of IFN-γ and IL-4 upon activation particularly by α-GalCer [211,212] and have diverse effects *in vivo* [213] that includes during myelin repair or remyelination.

Rendering iNKT-cells hyporesponsive or anergic to an endogenous GL is a novel insight into diseases manifesting aberrant iNKT-cell activation. Consequently this finding of GL ligand-driven anergy in MS has substantial implications. Anergy of MS circulating lymphocytes to the exogenous α-GalCer ligand [200] or to the endogenous polyacetylated-GalCers (FMCs) [201] has been observed [12,13,14] (Figure 1C). In healthy individuals iNKT cells expanded upon stimulation with α-GalCer or acetyl-β-GalCers accompanied by robust cytokine secretion [200,201] including secretion of cytokines associated with Th1 cells (IFN-γ), Th17 cells (IL-17, TNF-α) and both pro-inflammatory (IL-1β, IL-6, TNF-α) and anti-inflammatory responses (IL-10) while the anergy of human MS circulating lymphocytes is consistent with previous iNKT cell usage and probably reflects saturation of the iTCR by GL antigen. This suggests that lymphocyte reactivity and especially that involving inflammation, should be explored in regard to the failure or severe limitation of remyelination, the process that should repair inflammatory demyelination.

### 8.2. Promyelinating Antibodies and Potential for Remyelination in MS

The induction of remyelination within the demyelinated lesions of MS CNS is an important therapeutic objective for strategies that promote axon remyelination within a critical time are neuroprotective for MS patient benefit. Remyelination in an active MS lesion proceeds in an inflammatory setting with processes driving tissue damage as well as repair. However the restorative process is often limited suggesting that the numerous OPCs remaining within and at the rim of the MS plaque are blocked on the path to myelination [5,151,214,215]. Treatment to modulate this would be clinically valuable. Transplantation of cells capable of myelination and fostered by administration of growth, trophic and neuroprotective factors [216] is one possible way, and another is application of CNS reactive antibodies to promote remyelination [217,218].

Such antibodies can be synthesized *in situ* [219], in response to sensitization with CNS white matter [220,221] or identified from existing collections of human antibodies [216,222,223]. The intriguing observation that certain monoclonal antibodies IgM immunoglobulin induce remyelination in the toxin-mediated (lysolecithin [224,225]), autoimmune (EAE, [226]) and virus-induced Theiler’s murine encephalomyelitis (TMEV) [227] models of inflammatory demyelination resembling MS has been examined, and several mouse [228] and human [229] monoclonal antibodies identified. All of the remyelination-promoting IgMs (and no IgGs) that have been reported bind to oligodendrocytes and myelin. The rare IgMs with this capacity include the A2B5, O1, O4 and HNK-1 antibodies that bind GL antigens (GT3/9-O-Ac-GT3, GalCer, sGalCer and SGPG (sulfoglucuronyl determinant)) respectively. It appears that myelin lipid antigens are key antigens. A partial exception is a myelin-reactive anti-MOG antibody reported to promote remyelinating activity [218]. But for the latter we should note that the driving epitope for MOG might be the oligosaccharide. Other human antibodies, sHIgM42 and sHIgM12, that stimulate neuronal outgrowth appear to be directed against the oligosaccharides on gangliosides in neurons [230].

The human antibody promoting remyelination, designated as rHIgM22, was genetically engineered after being derived from the serum of patient with Waldenstrom’s macroglobulinemia: the cDNA encoding the antibody was isolated from circulating lymphocytes [229]. This recombinant version-rHIgM22-promotes remyelination admist the inflammatory demyelination of TMEV [231], and also of lysolecithin toxin-induced demyelination [217,224]. Furthermore, rHIgM22 binds to surface antigens on mouse, rat and human oligodendrocytes, where it has been shown to activate a distinct Ca^2+^ influx pathway [219] and also bind to lipid rafts inducing an anti-apoptotic signal correlated with suppression of caspase-3 activation [232]. Importantly, the human IgM promotes myelin repair in human in MS by 5 weeks following treatment with a single, very low dose (500 ng) injection [233] and with an accompanying decreased lesion load detectable by MRI [234]. This novel and unexpected benefit along with the availability of rHIgM22 antibodies, and their apparent pro-myelinating properties not only render them valuable therapeutic candidate for MS therapies but also point up another biological potential of lipids and GLs for immunological and pharmacological roles. Manipulating cellular and humoral facets of the immune system can promote endogenous CNS repair, and the lipid antigens may well contribute importantly to the repair by transplanted cells. Thus, there is reason for optimism for therapeutic advances in the foreseeable future.

### 8.3. Sphingoid Mediation of Inflammatory Demyelination and Treatment Options

FTY720 (2-amino-2-propane-1,3-diol hydrochloride; fingolimod) is a sphingolipid analogue that profoundly affects immune function. It was initially identified as a derivative of myriocin, a metabolite of the ascomycete fungus *Isaria sinclaria*. FTY720 is a pro-drug that is phosphorylated *in vivo* by the appropriate phosphoryl kinase sphingosine kinase 2 (SphK2) [235] to its biologically active from FTY720-phosphate (FTY720-P); a structural analog of sphingosine-1-phosphate (S1P). FTY720-P binds to four out of the five known S1P receptors (S1PR_1/3/4/5_) but not to S1PR_2_ to produce prolonged receptor down-regulation [236]. The mechanism of action for FTY720 includes effects upon lymphocyte trafficking initial activation and eventual down-regulation of S1PR_1_ to prevent lymphocyte egress from lymphoid tissue, thereby reducing auto-aggressive lymphocyte infiltration into sites of inflammation. The majority of circulating lymphocytes are sequestered in lymph nodes, thereby reducing peripheral lymphocyte counts and the recirculation of lymphocytes to the CNS [236]. Lymphocytes in secondary lymphoid organs and those remaining in blood continue to be functional. Interestingly, systemic exposure to FTY720-P, the pharmacologically active metabolite, appears to be administration route-dependent. Firstly, FTY720-P was quantifiable around its peak after oral administration, but not after intravenous infusion [235]. Secondly, oral administration of FTY720 produced a significantly lower blood lymphocyte content than intravenous administration [237]. These results suggest that: (i) SphK2 is active during the absorption process and/or first pass through the liver and (ii) at least part of the phosphorylation of FTY720 occurs in the liver where there is abundant expression of SphK2 [238]. Lymphocyte subsets bearing the surface markers CD3, CD4, CD8, CD16, CD20 (B cells), CD45RA (T naive cells), CD45RO (T memory cells) were reduced by FTY720, whereas the numbers of peripheral blood granulocytes, monocytes, eosinophils, erythrocytes, and platelets remain unchanged [239]. T-cell counts are decreased more than B-cell counts, and CD4^+^ cells are affected even more than CD8^+^ cells [240]. This relative sequestration of T cells may be particularly advantageous in treating MS, because CD4^+^ T-cells are considered to be major contributors to the pathophysiology. Moreover FTY720 directly affects T cell by enhancing function of Treg cells and inhibiting the differentiation of proinflammatory Th1 cells.

In addition to the immunological mechanism of FTY720 action, recent studies indicate that the CNS is also a site of action for FTY720 [241]. Lipophilic FTY720 crosses the BBB and down-regulates S1PR_1_ in neural cells and astrocytes to reduce astrogliosis, a phenomenon associated with neurodegeneration in MS. This effect might help restore gap-junctional communication of astrocytes with neurons and cells of BBB [242]. There is a link between FTY720 efficacy and a possible reduction in astrogliosis. FTY720 exposure potentiated lysolecthin-induced astrogliosis via S1PR_3_ and S1PR_5_ in spinal cord slices and reduced the accompanying demyelination.

Additional effects of FTY720 might result from modulation of S1PR_3_ in astrocytes and of S1PR_1_ and S1PR_5_ in oligodendrocytes. It is interesting that FTY720 levels are much higher in CNS than in blood and FTY720 deposition is mainly in CNS tissues rather than CSF [243]. In summary, FTY720 has effects on lymphocyte trafficking, on the development and function of T cell subsets, and on CNS cells, all of which might contribute to its immunosuppressive mechanisms.

FTY720 effectively ameliorates EAE [243,244,245,246,247]. FTY720-driven amelioration of local demyelination by inhibition of T cells infiltration in an EAE model may link this mode of anti-inflammatory prophylaxis to immune regulation [246]. FTY720 affects the *in vitro* biology of oligodendrocyte cell lineages (oligodendrocytes and OPCs) including survival, proliferation, migration, and differentiation, though we note that there are contradictory reports that may reflect FTY720 dosage or other variables [248,249]. Certainly these indications of how FTY720 reduces demyelination during EAE and/or MS [241] should be pursued for relevance to plaque healing and remyelination fostering.

Two phase 3 clinical trials including FREEDOMS (FTY720 *vs.* placebo) and TRANSFORMS (FTY720 *vs.* IFN-β, currently used drug for MS) have been completed. These studies supported FTY720 efficacy *versus* paired controls at dose 0.5 and 1.25 mg [250,251], and FTY720 was approved by the FDA in September 2010 and now in use as Gilenya. It is the first oral therapy for relapsing-remitting MS although its side effects can be daunting. Nevertheless, pharmacological blockade of the S1P-S1PR axis is promising for MS treatment.

## 9. Conclusions

Approaches directly targeting progenitors of myelinating oligodendrocytes may lead to improved long-term outcomes and delay accumulation of chronic demyelination and perhaps axonal transection in MS. They might combine currently-approved or include new, pending or forthcoming immunoregulators or other immune treatment. These strategies may need to be considered for existing therapies [8,252,253] or await successful clinical trial [253,254]. In some cases, these strategies have not yet been validated by successful clinical trial in human patients presumably for reasons of cost or rationales that have not been fully understood or compelling [255]. Potential pragmatic confounds include incomplete CNS access, actions on additional lineages, and species differences. Moreover, the relationship between demyelination and axonal transection remains unclear and this is particularly important since the latter is reported to underlie the permanent disability observed later in the disease course. Notwithstanding these difficulties, protective and regenerative approaches represent an increasingly important translational focus. Their successful development is a critical goal for MS research. Design of therapeutics targeting only the inflammatory component may be short-sighted. Instead, drugs attenuating multiple mechanisms of neuronal loss may have greater promise in the treatment of neurodegenerative diseases. Nonetheless, the volume of evidence linking pro-inflammatory cytokine involvement to neurodegeneration makes this an exciting field of study with undeniable clinical relevance.

## References

[1] Frohman E.M., Racke M.K., Raine C.S. (2006). Multiple sclerosis—The plaque and its pathogenesis. N. Engl. J. Med..

[2] Noseworthy J.H., Lucchinetti C., Rodriguez M., Weinshenker B.G. (2000). Multiple sclerosis. N. Engl. J. Med..

[3] Lucchinetti C., Bruck W., Parisi J., Scheithauer B., Rodriguez M., Lassmann H. (2000). Heterogeneity of multiple sclerosis lesions: Implications for the pathogenesis of demyelination. Ann. Neurol..

[4] Prineas J.W., Parratt J.D. (2012). Oligodendrocytes and the early multiple sclerosis lesion. Ann. Neurol..

[5] Franklin R.J. (2002). Why does remyelination fail in multiple sclerosis?. Nat. Rev. Neurosci..

[6] Trapp B.D., Peterson J., Ransohoff R.M., Rudick R., Mork S., Bo L. (1998). Axonal transection in the lesions of multiple sclerosis. N. Engl. J. Med..

[7] Chun J., Hartung H.P. (2010). Mechanism of action of oral fingolimod (FTY720) in multiple sclerosis. Clin. Neuropharmacol..

[8] Aharoni R. (2013). The mechanism of action of glatiramer acetate in multiple sclerosis and beyond. Autoimmun. Rev..

[9] Rosetti C.M., Maggio B., Oliveira R.G. (2008). The self-organization of lipids and proteins of myelin at the membrane interface. Molecular factors underlying the microheterogeneity of domain segregation. Biochim. Biophys. Acta.

[10] Norton W.T., Autilio L.A. (1966). The lipid composition of purified bovine brain myelin. J. Neurochem..

[11] Fewster M.E., Hirono H., Mead J.F. (1976). Lipid composition of myelin in multiple sclerosis. J. Neurol..

[12] Dasgupta S., Levery S.B., Hogan E.L. (2002). 3-*O*-acetyl-sphingosine-series myelin glycolipids: Characterization of novel 3-*O*-acetyl-sphingosine galactosylceramide. J. Lipid Res..

[13] Bennion B., Dasgupta S., Hogan E.L., Levery S.B. (2007). Characterization of novel myelin components 3-*O*-acetyl-sphingosine galactosylceramides by electrospray ionization Q-TOF MS and MS/CID-MS of Li^+^ adducts. J. Mass Spectrom..

[14] Podbielska M., Dasgupta S., Levery S.B., Tourtellotte W.W., Annuk H., Moran A.P., Hogan E.L. (2010). Novel myelin penta- and hexa-acetyl-galactosyl-ceramides: Structural characterization and immunoreactivity in cerebrospinal fluid. J. Lipid Res..

[15] Yu R.K., Ledeen R.W. (1972). Gangliosides of human, bovine, and rabbit plasma. J. Lipid Res..

[16] Xu L., Anchordoquy T.J. (2008). Cholesterol domains in cationic lipid/DNA complexes improve transfection. Biochim. Biophys. Acta.

[17] Xu Y., Ramu Y., Lu Z. (2008). Removal of phospho-head groups of membrane lipids immobilizes voltage sensors of K^+^ channels. Nature.

[18] Wheeler D., Bandaru V.V., Calabresi P.A., Nath A., Haughey N.J. (2008). A defect of sphingolipid metabolism modifies the properties of normal appearing white matter in multiple sclerosis. Brain.

[19] Podbielska M., Hogan E.L. (2009). Molecular and immunogenic features of myelin lipids: Incitants or modulators of multiple sclerosis?. Mult. Scler..

[20] Podbielska M., Levery S.B., Hogan E.L. (2011). The structural and functional role of myelin fast-migrating cerebrosides: Pathological importance in multiple sclerosis. Clin. Lipidol..

[21] Magavi S.S., Leavitt B.R., Macklis J.D. (2000). Induction of neurogenesis in the neocortex of adult mice. Nature.

[22] Richardson W.D., Kessaris N., Pringle N. (2006). Oligodendrocyte wars. Nat. Rev. Neurosci..

[23] Noll E., Miller R.H. (1993). Oligodendrocyte precursors originate at the ventral ventricular zone dorsal to the ventral midline region in the embryonic rat spinal cord. Development.

[24] Pringle N.P., Guthrie S., Lumsden A., Richardson W.D. (1998). Dorsal spinal cord neuroepithelium generates astrocytes but not oligodendrocytes. Neuron.

[25] Fu H., Qi Y., Tan M., Cai J., Takebayashi H., Nakafuku M., Richardson W., Qiu M. (2002). Dual origin of spinal oligodendrocyte progenitors and evidence for the cooperative role of Olig2 and Nkx2.2 in the control of oligodendrocyte differentiation. Development.

[26] Cai J., Qi Y., Hu X., Tan M., Liu Z., Zhang J., Li Q., Sander M., Qiu M. (2005). Generation of oligodendrocyte precursor cells from mouse dorsal spinal cord independent of Nkx6 regulation and Shh signaling. Neuron.

[27] Vallstedt A., Klos J.M., Ericson J. (2005). Multiple dorsoventral origins of oligodendrocyte generation in the spinal cord and hindbrain. Neuron.

[28] Kessaris N., Fogarty M., Iannarelli P., Grist M., Wegner M., Richardson W.D. (2006). Competing waves of oligodendrocytes in the forebrain and postnatal elimination of an embryonic lineage. Nat. Neurosci..

[29] Mekki-Dauriac S., Agius E., Kan P., Cochard P. (2002). Bone morphogenetic proteins negatively control oligodendrocyte precursor specification in the chick spinal cord. Development.

[30] Noble M., Fok-Seang J., Wolswijk G., Wren D. (1990). Development and regeneration in the central nervous system. Philos. Trans. R. Soc. Lond. B Biol. Sci..

[31] Fok-Seang J., Miller R.H. (1994). Distribution and differentiation of A2B5^+^ glial precursors in the developing rat spinal cord. J. Neurosci. Res..

[32] Richardson R.M., Holloway K.L., Bullock M.R., Broaddus W.C., Fillmore H.L. (2006). Isolation of neuronal progenitor cells from the adult human neocortex. Acta Neurochir..

[33] Baumann N., Pham-Dinh D. (2001). Biology of oligodendrocyte and myelin in the mammalian central nervous system. Physiol. Rev..

[34] Miller R.H. (2002). Regulation of oligodendrocyte development in the vertebrate CNS. Prog. Neurobiol..

[35] Nishiyama A. (2001). NG2 cells in the brain: A novel glial cell population. Hum. Cell.

[36] Dawson M.R., Polito A., Levine J.M., Reynolds R. (2003). NG2-expressing glial progenitor cells: An abundant and widespread population of cycling cells in the adult rat CNS. Mol. Cell. Neurosci..

[37] Fancy S.P., Chan J.R., Baranzini S.E., Franklin R.J., Rowitch D.H. (2011). Myelin regeneration: A recapitulation of development?. Annu. Rev. Neurosci..

[38] Huang J.K., Franklin R.J. (2011). Regenerative medicine in multiple sclerosis: Identifying pharmacological targets of adult neural stem cell differentiation. Neurochem. Int..

[39] Pfeiffer S.E., Warrington A.E., Bansal R. (1993). The oligodendrocyte and its many cellular processes. Trends Cell Biol..

[40] Lajtha A., Toth J., Fujimoto K., Agrawal H.C. (1977). Turnover of myelin proteins in mouse brain *in vivo*. Biochem. J..

[41] LeBaron F.N., Sanyal S., Jungalwala F.B. (1981). Turnover rate of molecular species of sphingomyelin in rat brain. Neurochem. Res..

[42] Baba H., Akita H., Ishibashi T., Inoue Y., Nakahira K., Ikenaka K. (1999). Completion of myelin compaction, but not the attachment of oligodendroglial processes triggers K^+^ channel clustering. J. Neurosci. Res..

[43] Simons M., Trajkovic K. (2006). Neuron-glia communication in the control of oligodendrocyte function and myelin biogenesis. J. Cell Sci..

[44] Ishibashi T., Dakin K.A., Stevens B., Lee P.R., Kozlov S.V., Stewart C.L., Fields R.D. (2006). Astrocytes promote myelination in response to electrical impulses. Neuron.

[45] Piaton G., Gould R.M., Lubetzki C. (2010). Axon-oligodendrocyte interactions during developmental myelination, demyelination and repair. J. Neurochem..

[46] Foster R.E., Connors B.W., Waxman S.G. (1982). Rat optic nerve: Electrophysiological, pharmacological and anatomical studies during development. Brain Res..

[47] Voyvodic J.T. (1989). Target size regulates calibre and myelination of sympathetic axons. Nature.

[48] Friede R.L. (1972). Control of myelin formation by axon caliber (with a model of the control mechanism). J. Comp. Neurol..

[49] Garbay B., Heape A.M., Sargueil F., Cassagne C. (2000). Myelin synthesis in the peripheral nervous system. Prog. Neurobiol..

[50] Saher G., Quintes S., Mobius W., Wehr M.C., Kramer-Albers E.M., Brugger B., Nave K.A. (2009). Cholesterol regulates the endoplasmic reticulum exit of the major membrane protein P0 required for peripheral myelin compaction. J. Neurosci..

[51] Chen Z.L., Yu W.M., Strickland S. (2007). Peripheral regeneration. Annu. Rev. Neurosci..

[52] Chan J.R., Watkins T.A., Cosgaya J.M., Zhang C., Chen L., Reichardt L.F., Shooter E.M., Barres B.A. (2004). NGF controls axonal receptivity to myelination by Schwann cells or oligodendrocytes. Neuron.

[53] Brinkmann B.G., Agarwal A., Sereda M.W., Garratt A.N., Muller T., Wende H., Stassart R.M., Nawaz S., Humml C., Velanac V. (2008). Neuregulin-1/ErbB signaling serves distinct functions in myelination of the peripheral and central nervous system. Neuron.

[54] Michailov G.V., Sereda M.W., Brinkmann B.G., Fischer T.M., Haug B., Birchmeier C., Role L., Lai C., Schwab M.H., Nave K.A. (2004). Axonal neuregulin-1 regulates myelin sheath thickness. Science.

[55] Black J.A., Waxman S.G., Smith K.J. (2006). Remyelination of dorsal column axons by endogenous Schwann cells restores the normal pattern of Nav1.6 and Kv1.2 at nodes of Ranvier. Brain.

[56] Zawadzka M., Rivers L.E., Fancy S.P., Zhao C., Tripathi R., Jamen F., Young K., Goncharevich A., Pohl H., Rizzi M. (2010). CNS-resident glial progenitor/stem cells produce Schwann cells as well as oligodendrocytes during repair of CNS demyelination. Cell Stem Cell.

[57] Rotshenker S. (2011). Wallerian degeneration: The innate-immune response to traumatic nerve injury. J. Neuroinflamm..

[58] Srivastava R., Aslam M., Kalluri S.R., Schirmer L., Buck D., Tackenberg B., Rothhammer V., Chan A., Gold R., Berthele A. (2012). Potassium channel KIR4.1 as an immune target in multiple sclerosis. N. Engl. J. Med..

[59] Lassmann H. (2008). Mechanisms of inflammation induced tissue injury in multiple sclerosis. J. Neurol. Sci..

[60] Frischer J.M., Bramow S., Dal-Bianco A., Lucchinetti C.F., Rauschka H., Schmidbauer M., Laursen H., Sorensen P.S., Lassmann H. (2009). The relation between inflammation and neurodegeneration in multiple sclerosis brains. Brain.

[61] Becher B., Bechmann I., Greter M. (2006). Antigen presentation in autoimmunity and CNS inflammation: How T lymphocytes recognize the brain. J. Mol. Med..

[62] Mukherjee S., Soe T.T., Maxfield F.R. (1999). Endocytic sorting of lipid analogues differing solely in the chemistry of their hydrophobic tails. J. Cell Biol..

[63] Mukherjee S., Maxfield F.R. (2000). Role of membrane organization and membrane domains in endocytic lipid trafficking. Traffic.

[64] Sugita M., Porcelli S.A., Brenner M.B. (1997). Assembly and retention of CD1b heavy chains in the endoplasmic reticulum. J. Immunol..

[65] Gadola S.D., Zaccai N.R., Harlos K., Shepherd D., Castro-Palomino J.C., Ritter G., Schmidt R.R., Jones E.Y., Cerundolo V. (2002). Structure of human CD1b with bound ligands at 2.3 A, a maze for alkyl chains. Nat. Immunol..

[66] Batuwangala T., Shepherd D., Gadola S.D., Gibson K.J., Zaccai N.R., Fersht A.R., Besra G.S., Cerundolo V., Jones E.Y. (2004). The crystal structure of human CD1b with a bound bacterial glycolipid. J. Immunol..

[67] Moody D.B., Ulrichs T., Muhlecker W., Young D.C., Gurcha S.S., Grant E., Rosat J.P., Brenner M.B., Costello C.E., Besra G.S. (2000). CD1c-mediated T-cell recognition of isoprenoid glycolipids in Mycobacterium tuberculosis infection. Nature.

[68] Shamshiev A., Gober H.J., Donda A., Mazorra Z., Mori L., de Libero G. (2002). Presentation of the same glycolipid by different CD1 molecules. J. Exp. Med..

[69] Steinman L. (1996). Multiple sclerosis: A coordinated immunological attack against myelin in the central nervous system. Cell.

[70] Engelhardt B., Ransohoff R.M. (2005). The ins and outs of T-lymphocyte trafficking to the CNS: Anatomical sites and molecular mechanisms. Trends Immunol..

[71] Alt C., Laschinger M., Engelhardt B. (2002). Functional expression of the lymphoid chemokines CCL19 (ELC) and CCL 21 (SLC) at the blood-brain barrier suggests their involvement in G-protein-dependent lymphocyte recruitment into the central nervous system during experimental autoimmune encephalomyelitis. Eur. J. Immunol..

[72] Columba-Cabezas S., Serafini B., Ambrosini E., Aloisi F. (2003). Lymphoid chemokines CCL19 and CCL21 are expressed in the central nervous system during experimental autoimmune encephalomyelitis: Implications for the maintenance of chronic neuroinflammation. Brain Pathol..

[73] Greter M., Heppner F.L., Lemos M.P., Odermatt B.M., Goebels N., Laufer T., Noelle R.J., Becher B. (2005). Dendritic cells permit immune invasion of the CNS in an animal model of multiple sclerosis. Nat. Med..

[74] Racke M.K., Scott D.E., Quigley L., Gray G.S., Abe R., June C.H., Perrin P.J. (1995). Distinct roles for B7-1 (CD-80) and B7-2 (CD-86) in the initiation of experimental allergic encephalomyelitis. J. Clin. Investig..

[75] Weinberg A.D., Wegmann K.W., Funatake C., Whitham R.H. (1999). Blocking OX-40/OX-40 ligand interaction *in vitro* and *in vivo* leads to decreased T cell function and amelioration of experimental allergic encephalomyelitis. J. Immunol..

[76] Wolswijk G., Balesar R. (2003). Changes in the expression and localization of the paranodal protein Caspr on axons in chronic multiple sclerosis. Brain.

[77] Coman I., Aigrot M.S., Seilhean D., Reynolds R., Girault J.A., Zalc B., Lubetzki C. (2006). Nodal, paranodal and juxtaparanodal axonal proteins during demyelination and remyelination in multiple sclerosis. Brain.

[78] Howell O.W., Palser A., Polito A., Melrose S., Zonta B., Scheiermann C., Vora A.J., Brophy P.J., Reynolds R. (2006). Disruption of neurofascin localization reveals early changes preceding demyelination and remyelination in multiple sclerosis. Brain.

[79] Trapp B.D., Nave K.A. (2008). Multiple sclerosis: An immune or neurodegenerative disorder?. Annu. Rev. Neurosci..

[80] Vickers J.C., King A.E., Woodhouse A., Kirkcaldie M.T., Staal J.A., McCormack G.H., Blizzard C.A., Musgrove R.E., Mitew S., Liu Y. (2009). Axonopathy and cytoskeletal disruption in degenerative diseases of the central nervous system. Brain Res. Bull..

[81] Aharoni R., Arnon R., Eilam R. (2005). Neurogenesis and neuroprotection induced by peripheral immunomodulatory treatment of experimental autoimmune encephalomyelitis. J. Neurosci..

[82] Liebetanz D., Merkler D. (2006). Effects of commissural de- and remyelination on motor skill behaviour in the cuprizone mouse model of multiple sclerosis. Exp. Neurol..

[83] Smith P.M., Jeffery N.D. (2006). Histological and ultrastructural analysis of white matter damage after naturally-occurring spinal cord injury. Brain Pathol..

[84] Prineas J.W., Barnard R.O., Kwon E.E., Sharer L.R., Cho E.S. (1993). Multiple sclerosis: Remyelination of nascent lesions. Ann. Neurol..

[85] Gupta R., Rowshan K., Chao T., Mozaffar T., Steward O. (2004). Chronic nerve compression induces local demyelination and remyelination in a rat model of carpal tunnel syndrome. Exp. Neurol..

[86] Raine C.S., Wu E. (1993). Multiple sclerosis: Remyelination in acute lesions. J. Neuropathol. Exp. Neurol..

[87] Hagemeier K., Bruck W., Kuhlmann T. (2012). Multiple sclerosis—Remyelination failure as a cause of disease progression. Histol. Histopathol..

[88] Nakahara J., Kanekura K., Nawa M., Aiso S., Suzuki N. (2009). Abnormal expression of TIP30 and arrested nucleocytoplasmic transport within oligodendrocyte precursor cells in multiple sclerosis. J. Clin. Invest..

[89] Patrikios P., Stadelmann C., Kutzelnigg A., Rauschka H., Schmidbauer M., Laursen H., Sorensen P.S., Bruck W., Lucchinetti C., Lassmann H. (2006). Remyelination is extensive in a subset of multiple sclerosis patients. Brain.

[90] Prineas J.W., Connell F. (1979). Remyelination in multiple sclerosis. Ann. Neurol..

[91] Redford E.J., Smith K.J., Gregson N.A., Davies M., Hughes P., Gearing A.J., Miller K., Hughes R.A. (1997). A combined inhibitor of matrix metalloproteinase activity and tumour necrosis factor-α processing attenuates experimental autoimmune neuritis. Brain.

[92] Byravan S., Foster L.M., Phan T., Verity A.N., Campagnoni A.T. (1994). Murine oligodendroglial cells express nerve growth factor. Proc. Natl. Acad. Sci. USA.

[93] Strelau J., Unsicker K. (1999). GDNF family members and their receptors: Expression and functions in two oligodendroglial cell lines representing distinct stages of oligodendroglial development. Glia.

[94] Wilkins A., Chandran S., Compston A. (2001). A role for oligodendrocyte-derived IGF-1 in trophic support of cortical neurons. Glia.

[95] Nishiyama A., Lin X.H., Giese N., Heldin C.H., Stallcup W.B. (1996). Co-localization of NG2 proteoglycan and PDGF α-receptor on O2A progenitor cells in the developing rat brain. J. Neurosci. Res..

[96] Pringle N.P., Mudhar H.S., Collarini E.J., Richardson W.D. (1992). PDGF receptors in the rat CNS: During late neurogenesis, PDGF α-receptor expression appears to be restricted to glial cells of the oligodendrocyte lineage. Development.

[97] Glezer I., Lapointe A., Rivest S. (2006). Innate immunity triggers oligodendrocyte progenitor reactivity and confines damages to brain injuries. FASEB J..

[98] Rhodes K.E., Raivich G., Fawcett J.W. (2006). The injury response of oligodendrocyte precursor cells is induced by platelets, macrophages and inflammation-associated cytokines. Neuroscience.

[99] Fancy S.P., Zhao C., Franklin R.J. (2004). Increased expression of Nkx2.2 and Olig2 identifies reactive oligodendrocyte progenitor cells responding to demyelination in the adult CNS. Mol. Cell. Neurosci..

[100] Smith K.J., Blakemore W.F., McDonald W.I. (1979). Central remyelination restores secure conduction. Nature.

[101] Oluich L.J., Stratton J.A., Xing Y.L., Ng S.W., Cate H.S., Sah P., Windels F., Kilpatrick T.J., Merson T.D. (2012). Targeted ablation of oligodendrocytes induces axonal pathology independent of overt demyelination. J. Neurosci..

[102] Gledhill R.F., Harrison B.M., McDonald W.I. (1973). Pattern of remyelination in the CNS. Nature.

[103] Blakemore W.F. (1974). Pattern of remyelination in the CNS. Nature.

[104] Ludwin S.K., Maitland M. (1984). Long-term remyelination fails to reconstitute normal thickness of central myelin sheaths. J. Neurol. Sci..

[105] Ghatak N.R., Leshner R.T., Price A.C., Felton W.L. (1989). 3rd Remyelination in the human central nervous system. J. Neuropathol. Exp. Neurol..

[106] Dupree J.L., Mason J.L., Marcus J.R., Stull M., Levinson R., Matsushima G.K., Popko B. (2004). Oligodendrocytes assist in the maintenance of sodium channel clusters independent of the myelin sheath. Neuron Glia Biol..

[107] Kornek B., Storch M.K., Weissert R., Wallstroem E., Stefferl A., Olsson T., Linington C., Schmidbauer M., Lassmann H. (2000). Multiple sclerosis and chronic autoimmune encephalomyelitis: A comparative quantitative study of axonal injury in active, inactive, and remyelinated lesions. Am. J. Pathol..

[108] Irvine K.A., Blakemore W.F. (2008). Remyelination protects axons from demyelination-associated axon degeneration. Brain.

[109] Charles P., Reynolds R., Seilhean D., Rougon G., Aigrot M.S., Niezgoda A., Zalc B., Lubetzki C. (2002). Re-expression of PSA-NCAM by demyelinated axons: An inhibitor of remyelination in multiple sclerosis?. Brain.

[110] Charles P., Hernandez M.P., Stankoff B., Aigrot M.S., Colin C., Rougon G., Zalc B., Lubetzki C. (2000). Negative regulation of central nervous system myelination by polysialylated-neural cell adhesion molecule. Proc. Natl. Acad. Sci. USA.

[111] Jakovcevski I., Mo Z., Zecevic N. (2007). Down-regulation of the axonal polysialic acid-neural cell adhesion molecule expression coincides with the onset of myelination in the human fetal forebrain. Neuroscience.

[112] Fewou S.N., Ramakrishnan H., Bussow H., Gieselmann V., Eckhardt M. (2007). Down-regulation of polysialic acid is required for efficient myelin formation. J. Biol. Chem..

[113] Franceschini I., Vitry S., Padilla F., Casanova P., Tham T.N., Fukuda M., Rougon G., Durbec P., Dubois-Dalcq M. (2004). Migrating and myelinating potential of neural precursors engineered to overexpress PSA-NCAM. Mol. Cell. Neurosci..

[114] Zhang H., Vutskits L., Calaora V., Durbec P., Kiss J.Z. (2004). A role for the polysialic acid-neural cell adhesion molecule in PDGF-induced chemotaxis of oligodendrocyte precursor cells. J. Cell Sci..

[115] Taveggia C., Feltri M.L., Wrabetz L. (2010). Signals to promote myelin formation and repair. Nat. Rev. Neurol..

[116] Jurynczyk M., Selmaj K. (2010). Notch: A new player in MS mechanisms. J. Neuroimmunol..

[117] D’Souza B., Miyamoto A., Weinmaster G. (2008). The many facets of Notch ligands. Oncogene.

[118] Wang S., Sdrulla A.D., diSibio G., Bush G., Nofziger D., Hicks C., Weinmaster G., Barres B.A. (1998). Notch receptor activation inhibits oligodendrocyte differentiation. Neuron.

[119] Stidworthy M.F., Genoud S., Li W.W., Leone D.P., Mantei N., Suter U., Franklin R.J. (2004). Notch1 and Jagged1 are expressed after CNS demyelination, but are not a major rate-determining factor during remyelination. Brain.

[120] Zhang Y., Argaw A.T., Gurfein B.T., Zameer A., Snyder B.J., Ge C., Lu Q.R., Rowitch D.H., Raine C.S., Brosnan C.F. (2009). Notch1 signaling plays a role in regulating precursor differentiation during CNS remyelination. Proc. Natl. Acad. Sci. USA.

[121] John G.R., Shankar S.L., Shafit-Zagardo B., Massimi A., Lee S.C., Raine C.S., Brosnan C.F. (2002). Multiple sclerosis: Re-expression of a developmental pathway that restricts oligodendrocyte maturation. Nat. Med..

[122] Peress N.S., Perillo E., Seidman R.J. (1996). Glial transforming growth factor (TGF)-beta isotypes in multiple sclerosis: Differential glial expression of TGF-β 1, 2 and 3 isotypes in multiple sclerosis. J. Neuroimmunol..

[123] Hu Q.D., Ang B.T., Karsak M., Hu W.P., Cui X.Y., Duka T., Takeda Y., Chia W., Sankar N., Ng Y.K. (2003). F3/contactin acts as a functional ligand for Notch during oligodendrocyte maturation. Cell.

[124] Cui X.Y., Hu Q.D., Tekaya M., Shimoda Y., Ang B.T., Nie D.Y., Sun L., Hu W.P., Karsak M., Duka T. (2004). NB-3/Notch1 pathway via Deltex1 promotes neural progenitor cell differentiation into oligodendrocytes. J. Biol. Chem..

[125] Derfuss T., Parikh K., Velhin S., Braun M., Mathey E., Krumbholz M., Kumpfel T., Moldenhauer A., Rader C., Sonderegger P. (2009). Contactin-2/TAG-1-directed autoimmunity is identified in multiple sclerosis patients and mediates gray matter pathology in animals. Proc. Natl. Acad. Sci. USA.

[126] Yiu G., He Z. (2006). Glial inhibition of CNS axon regeneration. Nat. Rev. Neurosci..

[127] Liu B.P., Fournier A., GrandPre T., Strittmatter S.M. (2002). Myelin-associated glycoprotein as a functional ligand for the Nogo-66 receptor. Science.

[128] Wang K.C., Koprivica V., Kim J.A., Sivasankaran R., Guo Y., Neve R.L., He Z. (2002). Oligodendrocyte-myelin glycoprotein is a Nogo receptor ligand that inhibits neurite outgrowth. Nature.

[129] Chen M.S., Huber A.B., van der Haar M.E., Frank M., Schnell L., Spillmann A.A., Christ F., Schwab M.E. (2000). Nogo-A is a myelin-associated neurite outgrowth inhibitor and an antigen for monoclonal antibody IN-1. Nature.

[130] Moreau-Fauvarque C., Kumanogoh A., Camand E., Jaillard C., Barbin G., Boquet I., Love C., Jones E.Y., Kikutani H., Lubetzki C. (2003). The transmembrane semaphorin Sema4D/CD100, an inhibitor of axonal growth, is expressed on oligodendrocytes and upregulated after CNS lesion. J. Neurosci..

[131] Benson M.D., Romero M.I., Lush M.E., Lu Q.R., Henkemeyer M., Parada L.F. (2005). Ephrin-B3 is a myelin-based inhibitor of neurite outgrowth. Proc. Natl. Acad. Sci. USA.

[132] Fournier A.E., GrandPre T., Strittmatter S.M. (2001). Identification of a receptor mediating Nogo-66 inhibition of axonal regeneration. Nature.

[133] Mi S., Lee X., Shao Z., Thill G., Ji B., Relton J., Levesque M., Allaire N., Perrin S., Sands B. (2004). LINGO-1 is a component of the Nogo-66 receptor/p75 signaling complex. Nat. Neurosci..

[134] Mi S., Miller R.H., Lee X., Scott M.L., Shulag-Morskaya S., Shao Z., Chang J., Thill G., Levesque M., Zhang M. (2005). LINGO-1 negatively regulates myelination by oligodendrocytes. Nat. Neurosci..

[135] Mi S. (2008). Troy/Taj and its role in CNS axon regeneration. Cytokine Growth Factor Rev..

[136] Laursen L.S., Chan C.W., Ffrench-Constant C. (2009). An integrin-contactin complex regulates CNS myelination by differential Fyn phosphorylation. J. Neurosci..

[137] Gentry J.J., Barker P.A., Carter B.D. (2004). The p75 neurotrophin receptor: Multiple interactors and numerous functions. Prog. Brain Res..

[138] Barker P.A. (2004). p75NTR is positively promiscuous: Novel partners and new insights. Neuron.

[139] Lee X., Yang Z., Shao Z., Rosenberg S.S., Levesque M., Pepinsky R.B., Qiu M., Miller R.H., Chan J.R., Mi S. (2007). NGF regulates the expression of axonal LINGO-1 to inhibit oligodendrocyte differentiation and myelination. J. Neurosci..

[140] Domeniconi M., Filbin M.T. (2005). Overcoming inhibitors in myelin to promote axonal regeneration. J. Neurol. Sci..

[141] Satoh J., Tabunoki H., Yamamura T., Arima K., Konno H. (2007). TROY and LINGO-1 expression in astrocytes and macrophages/microglia in multiple sclerosis lesions. Neuropathol. Appl. Neurobiol..

[142] Mi S., Hu B., Hahm K., Luo Y., Kam Hui E.S., Yuan Q., Wong W.M., Wang L., Su H., Chu T.H. (2007). LINGO-1 antagonist promotes spinal cord remyelination and axonal integrity in MOG-induced experimental autoimmune encephalomyelitis. Nat. Med..

[143] Rudick R.A., Mi S., Sandrock A.W. (2008). LINGO-1 antagonists as therapy for multiple sclerosis: *In vitro* and *in vivo* evidence. Expert Opin. Biol. Ther..

[144] Chun S.J., Rasband M.N., Sidman R.L., Habib A.A., Vartanian T. (2003). Integrin-linked kinase is required for laminin-2-induced oligodendrocyte cell spreading and CNS myelination. J. Cell Biol..

[145] Kramer E.M., Klein C., Koch T., Boytinck M., Trotter J. (1999). Compartmentation of Fyn kinase with glycosylphosphatidylinositol-anchored molecules in oligodendrocytes facilitates kinase activation during myelination. J. Biol. Chem..

[146] Relucio J., Tzvetanova I.D., Ao W., Lindquist S., Colognato H. (2009). Laminin alters fyn regulatory mechanisms and promotes oligodendrocyte development. J. Neurosci..

[147] Charles P., Tait S., Faivre-Sarrailh C., Barbin G., Gunn-Moore F., Denisenko-Nehrbass N., Guennoc A.M., Girault J.A., Brophy P.J., Lubetzki C. (2002). Neurofascin is a glial receptor for the paranodin/Caspr-contactin axonal complex at the axoglial junction. Curr. Biol..

[148] Colognato H., Galvin J., Wang Z., Relucio J., Nguyen T., Harrison D., Yurchenco P.D., Ffrench-Constant C. (2007). Identification of dystroglycan as a second laminin receptor in oligodendrocytes, with a role in myelination. Development.

[149] Chang A., Tourtellotte W.W., Rudick R., Trapp B.D. (2002). Premyelinating oligodendrocytes in chronic lesions of multiple sclerosis. N. Engl. J. Med..

[150] Wolswijk G. (1998). Chronic stage multiple sclerosis lesions contain a relatively quiescent population of oligodendrocyte precursor cells. J. Neurosci..

[151] Franklin R.J., Ffrench-Constant C. (2008). Remyelination in the CNS: From biology to therapy. Nat. Rev. Neurosci..

[152] Back S.A., Tuohy T.M., Chen H., Wallingford N., Craig A., Struve J., Luo N.L., Banine F., Liu Y., Chang A. (2005). Hyaluronan accumulates in demyelinated lesions and inhibits oligodendrocyte progenitor maturation. Nat. Med..

[153] Kuhlmann T., Miron V., Cui Q., Wegner C., Antel J., Bruck W. (2008). Differentiation block of oligodendroglial progenitor cells as a cause for remyelination failure in chronic multiple sclerosis. Brain.

[154] Zhao C., Li W.W., Franklin R.J. (2006). Differences in the early inflammatory responses to toxin-induced demyelination are associated with the age-related decline in CNS remyelination. Neurobiol. Aging.

[155] Blakemore W.F., Chari D.M., Gilson J.M., Crang A.J. (2002). Modelling large areas of demyelination in the rat reveals the potential and possible limitations of transplanted glial cells for remyelination in the CNS. Glia.

[156] Hanafy K.A., Sloane J.A. (2011). Regulation of remyelination in multiple sclerosis. FEBS Lett..

[157] Talbott J.F., Loy D.N., Liu Y., Qiu M.S., Bunge M.B., Rao M.S., Whittemore S.R. (2005). Endogenous Nkx2.2^+^/Olig2^+^ oligodendrocyte precursor cells fail to remyelinate the demyelinated adult rat spinal cord in the absence of astrocytes. Exp. Neurol..

[158] Miller R.H., Mi S. (2007). Dissecting demyelination. Nat. Neurosci..

[159] Franklin R.J., Kotter M.R. (2008). The biology of CNS remyelination: The key to therapeutic advances. J. Neurol..

[160] Nait-Oumesmar B., Picard-Riera N., Kerninon C., Decker L., Seilhean D., Hoglinger G.U., Hirsch E.C., Reynolds R., Baron-Van Evercooren A. (2007). Activation of the subventricular zone in multiple sclerosis: Evidence for early glial progenitors. Proc. Natl. Acad. Sci. USA.

[161] Nakahara T., Urabe K., Fukagawa S., Uchi H., Inaba K., Furue M., Moroi Y. (2005). Engagement of human monocyte-derived dendritic cells into interleukin (IL)-12 producers by IL-1β + interferon (IFN)-gamma. Clin. Exp. Immunol..

[162] Petermann F., Korn T. (2011). Cytokines and effector T cell subsets causing autoimmune CNS disease. FEBS Lett..

[163] Kroenke M.A., Carlson T.J., Andjelkovic A.V., Segal B.M. (2008). IL-12- and IL-23-modulated T cells induce distinct types of EAE based on histology, CNS chemokine profile, and response to cytokine inhibition. J. Exp. Med..

[164] Kang Z., Altuntas C.Z., Gulen M.F., Liu C., Giltiay N., Qin H., Liu L., Qian W., Ransohoff R.M., Bergmann C. (2010). Astrocyte-restricted ablation of interleukin-17-induced Act1-mediated signaling ameliorates autoimmune encephalomyelitis. Immunity.

[165] Huppert J., Closhen D., Croxford A., White R., Kulig P., Pietrowski E., Bechmann I., Becher B., Luhmann H.J., Waisman A. (2010). Cellular mechanisms of IL-17-induced blood-brain barrier disruption. FASEB J..

[166] Yu C., Argyropoulos G., Zhang Y., Kastin A.J., Hsuchou H., Pan W. (2008). Neuroinflammation activates Mdr1b efflux transport through NFkappaB: Promoter analysis in BBB endothelia. Cell Physiol. Biochem..

[167] Vela J.M., Molina-Holgado E., Arevalo-Martin A., Almazan G., Guaza C. (2002). Interleukin-1 regulates proliferation and differentiation of oligodendrocyte progenitor cells. Mol. Cell. Neurosci..

[168] Meeuwsen S., Persoon-Deen C., Bsibsi M., Ravid R., van Noort J.M. (2003). Cytokine, chemokine and growth factor gene profiling of cultured human astrocytes after exposure to proinflammatory stimuli. Glia.

[169] McFarland H.F., Martin R. (2007). Multiple sclerosis: A complicated picture of autoimmunity. Nat. Immunol..

[170] Lassmann H., Ransohoff R.M. (2004). The CD4-Th1 model for multiple sclerosis: A critical (correction of crucial) re-appraisal. Trends Immunol..

[171] Issazadeh S., Mustafa M., Ljungdahl A., Hojeberg B., Dagerlind A., Elde R., Olsson T. (1995). Interferon gamma, interleukin 4 and transforming growth factor beta in experimental autoimmune encephalomyelitis in Lewis rats: Dynamics of cellular mRNA expression in the central nervous system and lymphoid cells. J. Neurosci. Res..

[172] Nishihara M., Ogura H., Ueda N., Tsuruoka M., Kitabayashi C., Tsuji F., Aono H., Ishihara K., Huseby E., Betz U.A. (2007). IL-6–gp130–STAT3 in T cells directs the development of IL-17+ Th with a minimum effect on that of Treg in the steady state. Int. Immunol..

[173] Park H., Li Z., Yang X.O., Chang S.H., Nurieva R., Wang Y.H., Wang Y., Hood L., Zhu Z., Tian Q. (2005). A distinct lineage of CD4 T cells regulates tissue inflammation by producing interleukin 17. Nat. Immunol..

[174] Chen Z., Laurence A., Kanno Y., Pacher-Zavisin M., Zhu B.M., Tato C., Yoshimura A., Hennighausen L., O’Shea J.J. (2006). Selective regulatory function of Socs3 in the formation of IL-17-secreting T cells. Proc. Natl. Acad. Sci. USA.

[175] Mangan P.R., Harrington L.E., O’Quinn D.B., Helms W.S., Bullard D.C., Elson C.O., Hatton R.D., Wahl S.M., Schoeb T.R., Weaver C.T. (2006). Transforming growth factor-β induces development of the T(H)17 lineage. Nature.

[176] Matusevicius D., Kivisakk P., He B., Kostulas N., Ozenci V., Fredrikson S., Link H. (1999). Interleukin-17 mRNA expression in blood and CSF mononuclear cells is augmented in multiple sclerosis. Mult. Scler..

[177] Craner M.J., Hains B.C., Lo A.C., Black J.A., Waxman S.G. (2004). Co-localization of sodium channel Nav1.6 and the sodium-calcium exchanger at sites of axonal injury in the spinal cord in EAE. Brain.

[178] Craner M.J., Newcombe J., Black J.A., Hartle C., Cuzner M.L., Waxman S.G. (2004). Molecular changes in neurons in multiple sclerosis: Altered axonal expression of Nav1.2 and Nav1.6 sodium channels and Na^+^/Ca^2+^ exchanger. Proc. Natl. Acad. Sci. USA.

[179] Wu H.Y., Tomizawa K., Matsui H. (2007). Calpain-calcineurin signaling in the pathogenesis of calcium-dependent disorder. Acta Med. Okayama.

[180] Banik N.L., Chakrabarti A.K., Konat G.W., Gantt-Wilford G., Hogan E.L. (1992). Calcium-activated neutral proteinase (calpain) activity in C6 cell line: Compartmentation of mu and m calpain. J. Neurosci. Res..

[181] Chakrabarti A.K., Dasgupta S., Banik N.L., Hogan E.L. (1990). Ganglioside-modulated proteolysis by Ca^2+^-activated neutral proteinase (CANP): A role of glycoconjugates in CANP regulation. J. Neurochem..

[182] Chakrabarti A.K., Dasgupta S., Banik N.L., Hogan E.L. (1990). Regulation of the calcium-activated neutral proteinase (CANP) of bovine brain by myelin lipids. Biochim. Biophys. Acta.

[183] Cuzzocrea S., McDonald M.C., Mazzon E., Siriwardena D., Serraino I., Dugo L., Britti D., Mazzullo G., Caputi A.P., Thiemermann C. (2000). Calpain inhibitor I reduces the development of acute and chronic inflammation. Am. J. Pathol..

[184] Butler J.T., Samantaray S., Beeson C.C., Ray S.K., Banik N.L. (2009). Involvement of calpain in the process of Jurkat T cell chemotaxis. J. Neurosci. Res..

[185] Kanungo J., Zheng Y.L., Amin N.D., Pant H.C. (2009). Targeting Cdk5 activity in neuronal degeneration and regeneration. Cell. Mol. Neurobiol..

[186] Gill M.B., Perez-Polo J.R. (2009). Bax shuttling after rotenone treatment of neuronal primary cultures: Effects on cell death phenotypes. J. Neurosci. Res..

[187] Guyton M.K., Wingrave J.M., Yallapragada A.V., Wilford G.G., Sribnick E.A., Matzelle D.D., Tyor W.R., Ray S.K., Banik N.L. (2005). Upregulation of calpain correlates with increased neurodegeneration in acute experimental auto-immune encephalomyelitis. J. Neurosci. Res..

[188] Shields D.C., Avgeropoulos N.G., Banik N.L., Tyor W.R. (2000). Acute multiple sclerosis characterized by extensive mononuclear phagocyte infiltration. Neurochem. Res..

[189] Hassen G.W., Feliberti J., Kesner L., Stracher A., Mokhtarian F. (2006). A novel calpain inhibitor for the treatment of acute experimental autoimmune encephalomyelitis. J. Neuroimmunol..

[190] Bennett J.L., Stuve O. (2009). Update on inflammation, neurodegeneration, and immunoregulation in multiple sclerosis: Therapeutic implications. Clin. Neuropharmacol..

[191] Imam S.A., Guyton M.K., Haque A., Vandenbark A., Tyor W.R., Ray S.K., Banik N.L. (2007). Increased calpain correlates with Th1 cytokine profile in PBMCs from MS patients. J. Neuroimmunol..

[192] Smith A.W., Doonan B.P., Tyor W.R., Abou-Fayssal N., Haque A., Banik N.L. (2011). Regulation of Th1/Th17 cytokines and IDO gene expression by inhibition of calpain in PBMCs from MS patients. J. Neuroimmunol..

[193] Shields D.C., Banik N.L. (1999). Pathophysiological role of calpain in experimental demyelination. J. Neurosci. Res..

[194] Hassen G.W., Feliberti J., Kesner L., Stracher A., Mokhtarian F. (2008). Prevention of axonal injury using calpain inhibitor in chronic progressive experimental autoimmune encephalomyelitis. Brain Res..

[195] Das A., Guyton M.K., Matzelle D.D., Ray S.K., Banik N.L. (2008). Time-dependent increases in protease activities for neuronal apoptosis in spinal cords of lewis rats during development of acute experimental autoimmune encephalomyelitis. J. Neurosci. Res..

[196] Guyton M.K., Das A., Samantaray S., Wallace G.C., Butler J.T., Ray S.K., Banik N.L. (2010). Calpeptin Attenuated Inflammation, Cell Death, and Axonal Damage in Animal Model of Multiple Sclerosis. J. Neurosci. Res..

[197] Guyton M.K., Brahmachari S., Das A., Samantaray S., Inoue J., Azuma M., Ray S.K., Banik N.L. (2009). Inhibition of calpain attenuates encephalitogenicity of MBP-specific T cells. J. Neurochem..

[198] Adorini L. (2004). Antigen-based immunointervention in human autoimmune diseases. Trends Biotechnol..

[199] Jahng A.W., Maricic I., Pedersen B., Burdin N., Naidenko O., Kronenberg M., Koezuka Y., Kumar V. (2001). Activation of natural killer T cells potentiates or prevents experimental autoimmune encephalomyelitis. J. Exp. Med..

[200] O'Keeffe J., Gately C.M., Counihan T., Hennessy M., Leahy T., Moran A.P., Hogan E.L. (2008). T-cells expressing natural killer (NK) receptors are altered in multiple sclerosis and responses to alpha-galactosylceramide are impaired. J. Neurol. Sci..

[201] Gately C.M., Podbielska M., Counihan T., Hennessy M., Leahy T., Moran A.P., Hogan E.L., O’Keeffe J. (2013). Invariant Natural Killer T-cell anergy to endogenous myelin acetyl-glycolipids in multiple sclerosis. J. Neuroimmunol..

[202] Oldstone M.B. (2005). Molecular mimicry, microbial infection, and autoimmune disease: Evolution of the concept. Curr. Top. Microbiol. Immunol..

[203] Wucherpfennig K.W., Allen P.M., Celada F., Cohen I.R., De Boer R., Garcia K.C., Goldstein B., Greenspan R., Hafler D., Hodgkin P. (2007). Polyspecificity of T cell and B cell receptor recognition. Semin. Immunol..

[204] Jana A., Hogan E.L., Pahan K. (2009). Ceramide and neurodegeneration: Susceptibility of neurons and oligodendrocytes to cell damage and death. J. Neurol. Sci..

[205] Podbielska M., Krotkiewski H., Hogan E.L. (2012). Signaling and regulatory functions of bioactive sphingolipids as therapeutic targets in multiple sclerosis. Neurochem. Res..

[206] Taniguchi M., Tashiro T., Dashtsoodol N., Hongo N., Watarai H. (2010). The specialized iNKT cell system recognizes glycolipid antigens and bridges the innate and acquired immune systems with potential applications for cancer therapy. Int. Immunol..

[207] Porcelli S., Yockey C.E., Brenner M.B., Balk S.P. (1993). Analysis of T cell antigen receptor (TCR) expression by human peripheral blood CD4-8-α/β T cells demonstrates preferential use of several V β genes and an invariant TCR alpha chain. J. Exp. Med..

[208] Borg N., Holland M. (2008). The effect of glycosaminoglycans on rat gametes *in vitro* and the associated signal pathway. Reproduction.

[209] Scott-Browne J.P., Matsuda J.L., Mallevaey T., White J., Borg N.A., McCluskey J., Rossjohn J., Kappler J., Marrack P., Gapin L. (2007). Germline-encoded recognition of diverse glycolipids by natural killer T cells. Nat. Immunol..

[210] Hogan E.L., Podbielska M., O’Keeffe J. (2013). Implications of lymphocyte anergy to glycolipids in multiple sclerosis (MS): iNKT cells may mediate the MS infectious trigger. J. Clin. Cell. Immunol..

[211] Cui J., Shin T., Kawano T., Sato H., Kondo E., Toura I., Kaneko Y., Koseki H., Kanno M., Taniguchi M. (1997). Requirement for Valpha14 NKT cells in IL-12-mediated rejection of tumors. Science.

[212] Godfrey D.I., Rossjohn J. (2011). New ways to turn on NKT cells. J. Exp. Med..

[213] Ito K., Karasawa M., Kawano T., Akasaka T., Koseki H., Akutsu Y., Kondo E., Sekiya S., Sekikawa K., Harada M. (2000). Involvement of decidual Valpha14 NKT cells in abortion. Proc. Natl. Acad. Sci. USA.

[214] Foote A.K., Blakemore W.F. (2005). Inflammation stimulates remyelination in areas of chronic demyelination. Brain.

[215] Chari D.M., Blakemore W.F. (2002). New insights into remyelination failure in multiple sclerosis: Implications for glial cell transplantation. Mult. Scler..

[216] Ben-Hur T., Goldman S.A. (2008). Prospects of cell therapy for disorders of myelin. Ann. N. Y. Acad. Sci..

[217] Warrington A.E., Bieber A.J., Ciric B., Van Keulen V., Pease L.R., Mitsunaga Y., Paz Soldan M.M., Rodriguez M. (2001). Immunoglobulin-mediated CNS repair. J. Allergy Clin. Immunol..

[218] Warrington A.E., Rodriguez M. (2008). Remyelination-promoting human IgMs: Developing a therapeutic reagent for demyelinating disease. Curr. Top. Microbiol. Immunol..

[219] Paz Soldan M.M., Warrington A.E., Bieber A.J., Ciric B., Van Keulen V., Pease L.R., Rodriguez M. (2003). Remyelination-promoting antibodies activate distinct Ca^2+^ influx pathways in astrocytes and oligodendrocytes: Relationship to the mechanism of myelin repair. Mol. Cell. Neurosci..

[220] Lang W., Rodriguez M., Lennon V.A., Lampert P.W. (1984). Demyelination and remyelination in murine viral encephalomyelitis. Ann. N. Y. Acad. Sci..

[221] Huang D.W., McKerracher L., Braun P.E., David S. (1999). A therapeutic vaccine approach to stimulate axon regeneration in the adult mammalian spinal cord. Neuron.

[222] Rodriguez M., Lennon V.A., Benveniste E.N., Merrill J.E. (1987). Remyelination by oligodendrocytes stimulated by antiserum to spinal cord. J. Neuropathol. Exp. Neurol..

[223] Warrington A.E., Asakura K., Bieber A.J., Ciric B., Van Keulen V., Kaveri S.V., Kyle R.A., Pease L.R., Rodriguez M. (2000). Human monoclonal antibodies reactive to oligodendrocytes promote remyelination in a model of multiple sclerosis. Proc. Natl. Acad. Sci. USA.

[224] Bieber A.J., Warrington A., Asakura K., Ciric B., Kaveri S.V., Pease L.R., Rodriguez M. (2002). Human antibodies accelerate the rate of remyelination following lysolecithin-induced demyelination in mice. Glia.

[225] Pavelko K.D., van Engelen B.G., Rodriguez M. (1998). Acceleration in the rate of CNS remyelination in lysolecithin-induced demyelination. J. Neurosci..

[226] Miller D.J., Bright J.J., Sriram S., Rodriguez M. (1997). Successful treatment of established relapsing experimental autoimmune encephalomyelitis in mice with a monoclonal natural autoantibody. J. Neuroimmunol..

[227] Miller D.J., Sanborn K.S., Katzmann J.A., Rodriguez M. (1994). Monoclonal autoantibodies promote central nervous system repair in an animal model of multiple sclerosis. J. Neurosci..

[228] Asakura K., Miller D.J., Murray K., Bansal R., Pfeiffer S.E., Rodriguez M. (1996). Monoclonal autoantibody SCH94.03, which promotes central nervous system remyelination, recognizes an antigen on the surface of oligodendrocytes. J. Neurosci. Res..

[229] Mitsunaga Y., Ciric B., Van Keulen V., Warrington A.E., Paz Soldan M., Bieber A.J., Rodriguez M., Pease L.R. (2002). Direct evidence that a human antibody derived from patient serum can promote myelin repair in a mouse model of chronic-progressive demyelinating disease. FASEB J..

[230] Rodriguez M., Warrington A.E., Pease L.R. (2009). Invited Article: Human natural autoantibodies in the treatment of neurologic disease. Neurology.

[231] Duncan I.D. (1996). Glial cell transplantation and remyelination of the central nervous system. Neuropathol. Appl. Neurobiol..

[232] Howe C.L., Bieber A.J., Warrington A.E., Pease L.R., Rodriguez M. (2004). Antiapoptotic signaling by a remyelination-promoting human antimyelin antibody. Neurobiol. Dis..

[233] Warrington A.E., Bieber A.J., Ciric B., Pease L.R., Van Keulen V., Rodriguez M. (2007). A recombinant human IgM promotes myelin repair after a single, very low dose. J. Neurosci. Res..

[234] Pirko I., Ciric B., Gamez J., Bieber A.J., Warrington A.E., Johnson A.J., Hanson D.P., Pease L.R., Macura S.I., Rodriguez M. (2004). A human antibody that promotes remyelination enters the CNS and decreases lesion load as detected by T2-weighted spinal cord MRI in a virus-induced murine model of MS. FASEB J..

[235] Zemann B., Kinzel B., Muller M., Reuschel R., Mechtcheriakova D., Urtz N., Bornancin F., Baumruker T., Billich A. (2006). Sphingosine kinase type 2 is essential for lymphopenia induced by the immunomodulatory drug FTY720. Blood.

[236] Matloubian M., Lo C.G., Cinamon G., Lesneski M.J., Xu Y., Brinkmann V., Allende M.L., Proia R.L., Cyster J.G. (2004). Lymphocyte egress from thymus and peripheral lymphoid organs is dependent on S1P receptor 1. Nature.

[237] Kovarik J.M., Hartmann S., Bartlett M., Riviere G.J., Neddermann D., Wang Y., Port A., Schmouder R.L. (2007). Oral-intravenous crossover study of fingolimod pharmacokinetics, lymphocyte responses and cardiac effects. Biopharm. Drug Dispos..

[238] Liu H., Sugiura M., Nava V.E., Edsall L.C., Kono K., Poulton S., Milstien S., Kohama T., Spiegel S. (2000). Molecular cloning and functional characterization of a novel mammalian sphingosine kinase type 2 isoform. J. Biol. Chem..

[239] Kahan B.D., Karlix J.L., Ferguson R.M., Leichtman A.B., Mulgaonkar S., Gonwa T.A., Skerjanec A., Schmouder R.L., Chodoff L. (2003). Pharmacodynamics, pharmacokinetics, and safety of multiple doses of FTY720 in stable renal transplant patients: A multicenter, randomized, placebo-controlled, phase I study. Transplantation.

[240] Budde K., Schmouder R.L., Nashan B., Brunkhorst R., Lücker P.W., Mayer T., Brookman L., Nedelman J., Skerjanec A., Bohler T., Neumayer H.H. (2003). Pharmacodynamics of single doses of the novel immunosuppressant FTY720 in stable renal transplant patients. Am. J. Transplant..

[241] Lee C.W., Choi J.W., Chun J. (2010). Neurological S1P signaling as an emerging mechanism of action of oral FTY720 (fingolimod) in multiple sclerosis. Arch. Pharm. Res..

[242] Brinkmann V. (2009). FTY720 (fingolimod) in Multiple Sclerosis: Therapeutic effects in the immune and the central nervous system. Br. J. Pharmacol..

[243] Foster C.A., Howard L.M., Schweitzer A., Persohn E., Hiestand P.C., Balatoni B., Reuschel R., Beerli C., Schwartz M., Billich A. (2007). Brain penetration of the oral immunomodulatory drug FTY720 and its phosphorylation in the central nervous system during experimental autoimmune encephalomyelitis: Consequences for mode of action in multiple sclerosis. J. Pharmacol. Exp. Ther..

[244] Fujino M., Funeshima N., Kitazawa Y., Kimura H., Amemiya H., Suzuki S., Li X.K. (2003). Amelioration of experimental autoimmune encephalomyelitis in Lewis rats by FTY720 treatment. J. Pharmacol. Exp. Ther..

[245] Webb M., Tham C.S., Lin F.F., Lariosa-Willingham K., Yu N., Hale J., Mandala S., Chun J., Rao T.S. (2004). Sphingosine 1-phosphate receptor agonists attenuate relapsing-remitting experimental autoimmune encephalitis in SJL mice. J. Neuroimmunol..

[246] Kataoka H., Sugahara K., Shimano K., Teshima K., Koyama M., Fukunari A., Chiba K. (2005). FTY720, sphingosine 1-phosphate receptor modulator, ameliorates experimental autoimmune encephalomyelitis by inhibition of T cell infiltration. Cell. Mol. Immunol..

[247] Balatoni B., Storch M.K., Swoboda E.M., Schonborn V., Koziel A., Lambrou G.N., Hiestand P.C., Weissert R., Foster C.A. (2007). FTY720 sustains and restores neuronal function in the DA rat model of MOG-induced experimental autoimmune encephalomyelitis. Brain Res. Bull..

[248] Miron V.E., Hall J.A., Kennedy T.E., Soliven B., Antel J.P. (2008). Cyclical and dose-dependent responses of adult human mature oligodendrocytes to fingolimod. Am. J. Pathol..

[249] Miron V.E., Jung C.G., Kim H.J., Kennedy T.E., Soliven B., Antel J.P. (2008). FTY720 modulates human oligodendrocyte progenitor process extension and survival. Ann. Neurol..

[250] Kappos L., Radue E.W., O'Connor P., Polman C., Hohlfeld R., Calabresi P., Selmaj K., Agoropoulou C., Leyk M., Zhang-Auberson L. (2010). A placebo-controlled trial of oral fingolimod in relapsing multiple sclerosis. N. Engl. J. Med..

[251] Cohen J.A., Barkhof F., Comi G., Hartung H.P., Khatri B.O., Montalban X., Pelletier J., Capra R., Gallo P., Izquierdo G. (2010). Oral fingolimod or intramuscular interferon for relapsing multiple sclerosis. N. Engl. J. Med..

[252] Gold R., Giovannoni G., Selmaj K., Havrdova E., Montalban X., Radue E.W., Stefoski D., Robinson R., Riester K., Rana J. (2013). Daclizumab high-yield process in relapsing-remitting multiple sclerosis (SELECT): A randomised, double-blind, placebo-controlled trial. Lancet.

[253] Klotz L., Wiendl H. (2013). Monoclonal antibodies in neuroinflammatory diseases. Expert Opin. Biol. Ther..

[254] Ali R., Nicholas R.S., Muraro P.A. (2013). Drugs in development for relapsing multiple sclerosis. Drugs.

[255] Huang J.K., Franklin R.J. (2012). Current status of myelin replacement therapies in multiple sclerosis. Prog. Brain Res..

